# Selection of Reference Genes for Gene Expression Normalization in *Peucedanum praeruptorum* Dunn under Abiotic Stresses, Hormone Treatments and Different Tissues

**DOI:** 10.1371/journal.pone.0152356

**Published:** 2016-03-29

**Authors:** Yucheng Zhao, Jun Luo, Sheng Xu, Wei Wang, Tingting Liu, Chao Han, Yijun Chen, Lingyi Kong

**Affiliations:** 1 State Key Laboratory of Natural Medicines, Department of Natural Medicinal Chemistry, China Pharmaceutical University, Nanjing, Jiangsu, P. R. China; 2 Institute of Botany, Jiangsu Province and Chinese Academy of Sciences, Nanjing, Jiangsu, P. R. China; Jilin University, CHINA

## Abstract

*Peucedanum praeruptorum* Dunn is one of the main traditional Chinese medicines producing coumarins and plenty of literatures are focused on the biosynthesis of coumarins. Quantitative real-time reverse transcription PCR (qRT-PCR) is a widely used method in studying the biosynthesis pathway and the selection of reference genes plays a crucial role in accurate normalization. To facilitate biosynthesis study of coumarins, twelve candidate reference genes were selected from the transcriptome database of *P*. *praeruptorum* according to previous studies. Then, BestKeeper, geNoFrm and NormFinder were used for selecting stably expressed reference genes in different tissues and under various stress treatments. The results indicated that, among the twelve candidate reference genes, the *SAND* family protein (*SAND*), actin 2 (*ACT2*), ubiquitin-conjugating enzyme 9 (*UBC9*), protein phosphatase 2A gene (*PP2A*) and polypyrimidine tract-binding protein (PTBP1) were the most stable reference genes under different experimental treatments, while glyceraldehyde 3-phosphate dehydrogenase (*GAPDH*) and tubulin beta-6 (*TUB6*) were the least stable genes. In addition, the suitability of *SAND*, *TIP41*-like protein (*TIP41*), *UBC9*, *ACT2*, *TUB6* and their combination as reference genes were confirmed by normalizing the expression of 1-aminocyclopropane-1-carboxylate oxidase (*ACO*) in different treatments. This work is the first survey of the stability of reference genes in *P*. *praeruptorum* and provides guidelines to obtain more accurate qRT-PCR results in *P*. *praeruptorum* and other plant species.

## Introduction

Plant secondary metabolites have various biological activities and pharmacological importance to human beings. As important members of plant secondary metabolites, coumarin compounds have received continuous attention, at the same time their chemical structures and biological activities have been well investigated [1–3]. Traditionally, harvesting of cultivated plants or gathering of wild plants and then solvent extraction remains the method of choice to secure the supply of medical compounds, while it may be a big challenge to surrounding environments. In addition, low abundance and season- or region-dependent sourcing also restrict its widespread use and availability [4,5]. Engineering microorganisms to produce natural compounds has shown great promise in tackling these problems by reassembly plant-specific biosynthetic pathways in microbial systems and plenty of efforts have been succeeded [6–8]. However, little is known in their biosynthetic mechanisms although the phenylpropanoid-derived coumarin compound biosynthetic pathway determined half a century ago with the help of tracer-feeding experiments. Recently, there are also plenty of literatures focused on the study of biosynthesis of coumarins in plants [9–14].

As the popular usage of next-generation-sequencing technology (NGS), including Roche/454 and Illumina HiSeq platforms, comprehensive analysis of gene expression has been successfully achieved in plant functional genomics predication or confirmation [15–17]. And, another technology, qRT-PCR is also widely used for gene expression studies owing to its quantitative accuracy, high sensitivity and high-throughput capabilities [14,18–20]. However, the results are inevitably affected by sample amount, RNA integrity, primer design, cDNA quality, as well as the PCR efficiency [21–23]. To eliminate the discrepancies and ensure the accuracy and reliability of the experimental results, a suitability internal reference is necessary. There are also reports suggesting that at least three reference genes need to be merged to normalize the results of qRT-PCR [18,22,24]. In addition, considering the gene expression level may differ from tissues to tissues, species to species, or even in different experimental conditions [25–27], selection of suitable reference genes specific for a given experimental design or species seems outmost important.

In recent years, with the progress in NGS and increasing awareness of the importance of suitable reference genes in normalization, plenty of literatures have been published with focus on the identification and selection of reference genes, including human [28,29], insects [30], bacteria [31], animals [32] and plants [33,34]. Besides, several statistical algorithms, namely NormFinder, BestKeeper, RefFinder, geNorm and deltaCt method, have been developed, which makes it easily to determine the most stable reference genes [23,24,35–37]. However, there are no studies concentrated on the selection of suitable reference genes for *P*. *praeruptorum*, in spite of its important medical usage and increasingly studies focusing on the biosynthesis pathway of coumarins [9–13]. Hence, it is useful and urgent for us to identify and select suitable reference genes for future molecular studies using qRT-PCR.

Traditionally, *GAPDH*, elongation factor-1α (*EF-1α*), tubulin β-chain (*β-TUB*), polyubiquitin (*UBQ*), 18S ribosomal RNA (18S rRNA), and *ACT* are selected as reference genes for their housekeeping roles in basic cellular processes [22,38,39]. However, the stability of these housekeeping genes restricts in special experimental designs or treatments. And, the different materials tend to have different stable genes. For instance, *ACT* and *TUB* are the most stable genes identified among all sample groups in carrot leaves with abiotic stresses and hormone stimuli, while *GAPDH* displays the maximum stability under most kinds of single stresses [40]. *EF-1α*, *β-TUB*, cyclophilin (*CYP*) and eukaryotic translation initiation factor 4α (*eIF-4α*) are the most reliable reference genes in maize but *ACT7* and *ACT101* are assessed as the most suitable sets for normalization in *Oxytropis ochrocephala* Bunge [22,41]. Besides, other studies indicate that some new reference genes, such as *eIF-4α*, *SAND* and *TIP41* [40,42–43], can also be selected as candidate reference genes for normalization. They also display a stably expressed behavior across different tissues and under various experimental treatments [40,42–43]. Hence, in this study, *TIP41*, *TUB6*, *SAND*, *ACT2*, *CYP2*, *GAPDH*, nuclear cap binding protein 20 (*NCBP20*), *eIF-4α*, *EF-1α*, *PP2A*, *UBC9* and *PTBP1* were selected from the transcriptome database of *P*. *praeruptorum* to investigate and choose the suitable reference genes for normalization [14]. To determine the stability of reference genes, different experimental treatments, including osmotic stress (polyethylene glycol, PEG), salt stress (NaCl), oxidative stress (H_2_O_2_), mental stress (CuSO_4_), hormones (methyl jasmonate (MeJA) and salicylic acid (SA)), cold (4°C) and heat (42°C) stress, and tissue pattern, were conducted to ensure that the reference genes selected in this study could be used in various experimental treatments designed for qRT-PCR [44,45]. To analysis the raw data, three kinds of Excel-based software, geNorm [24], NormFinder [23] and BestKeeper [35] were employed according to the manufacturer’s procedures. The results indicated that *SAND*, *ACT2*, *UBC9*, *PP2A* and *PTBP1* were the most stable reference genes, while *GAPDH* and *TUB6* were the least stable genes. In addition, geNorm was used to determine the optimal numbers of the reference genes required for accurate normalization by pairwise variation (Vn/Vn+1) [24] and the results indicated that under most groups, selection of two reference genes could be sufficient for normalization. To confirm the suitability of selected reference genes, *SAND*, *TIP41*, *UBC9*, *ACT2*, *TUB6* and their combination were used as reference genes to normalize the expression of *ACO* in different treatments. The results showed a good correlation with the stability rank revealed by the method of predication used in this study, which proved that the reference genes identified in this work were desirable. To the best of our knowledge, this work is the first survey of the stability of reference genes in *P*. *praeruptorum*, which would provide the basis for further research in exploring the metabolism and regulation mechanism to environment stresses, especially the secondary metabolism involved in biosynthesis of coumarin compounds [6,9,11].

## Materials and Methods

### Plant sample preparation and treatment

One-year-old *P*. *praeruptorum* materials were collected from the fields of Ningguo City, Anhui Province, China (longitude: 118.95E, latitude: 30.62N). The field of *P*. *praeruptorum* was a private land and the owner of the land had given permission to us for using the plant to conduct the study. Then the plants were transplanted in plastic basins containing a mixture of vermiculite, perlite, and peat moss at a ratio of 1:1:1. The plants were grown in a greenhouse with a long photoperiod (16 h light and 8 h dark) at 25°C, 40–65% relative humidity and 3000 lux of light intensity until use. For drought treatment, plants were subjected to 200 mL of 25% PEG 6000 per day for one week. In salt stress treatment, about 200 mL (600 mM) of NaCl was applied for seven days. For cold and heat shock treatments, plants were transferred to a greenhouse with the temperature of 4°C and 42°C for 48 h, respectively. For hormone treatments, MeJA (25 mM) and SA (5 mM) were imposed for 6 h according to the method described before [43]. Heavy metal stress was conducted using 500 mM copper sulfate (CuSO_4_) for 24 h and oxidative stress was carried out with 50 mM H_2_O_2_ for 24 h. All of the treatments were processed in three biological replicates. The plants without treatments were collected as control and the harvested samples were washed with MINIQ-filtered water and frozen and stored in liquid nitrogen prior to RNA isolation.

### Selection and validation of candidate reference genes and primer design

According to previous studies [14,22,38–42], twelve candidate genes (*ACT2*, *CYP2*, *EF-1α*, *eIF-4α*, *GAPDH*, *NCBP20*, *PP2A*, *PTBP1*, *SAND*, *TIP41*, *TUB6* and *UBC9*) were used to identify the most stable reference genes of *P*. *praeruptorum* in different treatments and their totally information was listed in Table 1. To meet this goal, the protein sequences of the twelve *Arabidopsis* genes were selected from TAIR database (http://www.arabidopsis.org) to set as the templates. And then, a local BLAST was conducted using the tblastn program in Bioedit Sequence Alignment Editor. The nucleotide sequences were also downloaded to find the intron for primer design using Primer 5. The primers listed in Table 1 were optimized for the primer lengths of 22 bp, GC content of 44% to 60% and amplification lengths from 84 bp to 170 bp. To validate the reliability of the selected candidate reference genes, the relative expression profiles of *ACO* were measured and normalized with the most and least stable reference genes under different experimental designs. The samples without treatments were used as control. The relative expression data was calculated by the 2^-ΔΔCT^ method [46] and presented as relative expression level. Three biological and technical replicates were used to obtain the qPCR data and the raw data were listed in S1 Table.

**Table 1:** Information of candidate reference genes selected for evaluation of expression stability in *P*. *praeruptorum*.

Gene Name	Description	Arabidopsis homolog locus	Primer: forward/reverse(5’-3’)	Length (bp)	PCR efficiency	TBLASTN Score	E-value ID (%)
***TIP41***	TIP41-like protein	At4g34270	F:TTGACTGCACTTGCATCAAAAG R:CGACACTCCACTATCAGCCAAT	87	97.88	422/1e-119	206/287 (71%)
***TUB6***	Tubulin beta-6	At5g12250	F:GGTGCTGGTAATAATTGGGCCA R:CCCATTCCAGATCCAGTTCCAC	158	101.69	870/0.0	418/443 (94%)
***SAND***	SAND family protein	At2g28390	F:ACAGAAGAGCCTCATGAATCCC R:CAAGCAAAGGCGTCATATCAAA	98	97.48	580/1e-166	312/517 (60%)
***ACT2***	Actin 2	At3g18780	F:TTTCACTATATGCCAGTGGTCG R:CTTCGTAGATCGGGACAGTGTG	84	92.34	536/1e-153	259/280 (92%)
***CYP2***	Cyclophilin 2	At4g33060	F:CGTTCAGCTCTGTCTCGAAGGT R:TAAGGCGGGAATGGAACTCATC	155	92.42	600/1e-172	310/511 (60%)
***GAPDH***	Glyceraldehyde 3-phosphate dehydrogenase	At1g42970	F:GGTCATGGGAGATGACATGGTC R:CAGGGTTTGTCTCGCAAAAATC	164	95.82	699/0.0	345/374 (92%)
***NCBP20***	Nuclear cap binding protein 20	At5g44200	F:GGCCAGGTACGCGATGAATATC R:CCCTTTGTGCTTCCAACTCCTT	91	100.38	429/1e-121	200/256 (78%)
***eIF-4α***	Eukaryotic translation initiation factor 4α-1	At3g13920	F:GCGCAGTCGTGACCACACAGTT R:TTGCTGCACATCAATACCACGG	145	109.23	767/0.0	383/413 (92%)
***EF-1α***	Elongation factor 1-α	At1g07940	F:CAAGCAGATGATCTGTTGCTGC R:TCATGTTGTCACCCTCGAATCC	170	97.29	856/0.0	419/446 (93%)
***PP2A***	Protein phosphatase 2A gene	At1g59830	F:CATGGAGGGCTTTCACCATCTC R:CGGTCGTCTGGATCAGACCATA	119	102.79	591/1e-169	276/293 (94%)
***UBC9***	Ubiquitin-conjugating enzyme 9	At4g2796	F:CTCGAAGCGGATCTTGAAGGAG R:CACCCGCATATGGACTGTCAGG	137	105.68	304/1e-83	142/148 (95%)
***PTBP1***	Polypyrimidine tract-binding protein	At3g01150	F:CCAGAACATGTTGGTTCTTGCA R:TCCCTCAATTGCAGTTGCATTA	135	109.65	590/1e-169	262/440(59%)

### Total RNA, DNA extraction and cDNA synthesis

According to the manufacturer’s recommendations, TransZol Plant reagent (TransGen Biotech, Beijing, China) was used to isolate the total RNA, then Spectramax plus384 enzyme-labeling instrument (Molecular Devices, Sunnyvale, USA) and agarose gel electrophoresis were used to assess the quantity and quality of the RNA. To eliminate DNA contamination, the samples were treated with DNase I (Takara, Dalian, China) according to the manufacturer’s protocol. For qRT-PCR experiments, cDNA was synthesized in a 20 μl reaction volume using a quantity of 1 μg total RNA (Takara, Dalian, China). Then, the plasmid DNA containing each gene was diluted 10-fold with nuclease-free water for qPCR analysis and continue diluted (10^2^×, 10^3^×, 10^4^×, 10^5^×, 10^6^× and 10^7^×dilutions) for determining the amplification efficiency (E) and correlation coefficient (R^2^). To achieve the aim of amplification of the genomic DNA of target reference genes, EasyPure Plant Genomic DNA Kit (TransGen Biotech, Beijing, China) was used to isolate the total genomic DNA according to the manufacturer’s protocol.

### RT-PCR and qPCR analysis

To verify the specificity of the target genes, PCR was performed using cDNA as a template. To verify the primer design containing intron, an analogous PCR procedure was conducted using total genomic DNA as a template. And then, all PCR products were examined by 1% (w/v) agarose gel electrophoresis. To further confirm the PCR products and obtain the nucleotide sequences of the target genes, the PCR products were inserted into pMD19-T Vector (Takara, Dalian, China) for sequencing and the results are listed in S2 Table. qRT-PCR analysis was conducted using LightCycler 480 (Roche Molecular Biochemicals, Mannheim, Germany) and AceQ qPCR SYBR Green Master Mix (Vazyme, Nangjing, China). Reaction mixtures contained 10 μL AceQ qPCR SYBR Green Master Mix, 2 μL diluted cDNA, 0.4 μM primer and ddH_2_O in a total volume of 20 μL. According to the procedures of specification, the following amplification conditions were applied: 1 cycle of 95°C for 5 min, 40 cycles of 95°C for 10 sec and then 60°C for 30 sec, followed by 1 cycle of 95°C for 15 sec, 60°C for 60 sec and 95°C for 15 sec. The RNase-free water was used as a control and three technical replicates were contained in each plate.

### Gene expression stability analysis

To analyze the expression stability of candidate reference genes, geNorm [24], NormFinder [23] and BestKeeper [35] were used according to the experimental design and manufacturer’s procedures. For geNorm and NormFinder analysis, the raw Cp values under different experimental designs were transformed into relative quantities using the formula 2^-ΔCT^ (ΔCT = each corresponding Ct value—lowest Ct value and Cp is another name of Ct) and then imported to geNorm to analyze gene expression stability value (M). Similar to geNorm, NormFinder was further used to investigate the expression stability values (M) for each gene and the pairwise variation (V) of that gene with other reference genes. The reference gene with the highest M value is considered as the most unstable gene while the lowest M value indicated the most stable gene. BestKeeper analysis was based on the untransformed Cp values and using coefficient of variance (CV) and the standard deviation (SD) of the Cp to evaluate the stability of reference genes. It was also used to rank the candidates from the most to least stably expressed genes. By the combination of the three kinds of Microsoft Excel-based software, we could easily rank the expression stability of reference genes in different experimental sets.

### Statistical analysis

Three biological and technical replicates were used to obtain the qPCR data. Unless the special comments, all data were presented as mean ± standard error of the mean (SEM). Statistical analyses were performed using the Student’s t-test. Graphs were generated using OriginPro 8 (OriginLab Corporation, Northampton, MA, USA). Data analysis was performed using geNorm [24], NormFinder [23] and BestKeeper [35] according to the manufacturer’s procedures.

## Results

### Selection of candidate reference genes, amplification specification and PCR efficiency

According to previous studies [14,22,38–42], the protein sequences of the twelve genes (*TIP41*, *TUB6*, *SAND*, *ACT2*, *CYP2*, *GAPDH*, *BCBP20*, *eIF-4α*, *EF-1α*, *PP2A*, *UBC9* and *PTBP1*) of *Arabidopsis* were selected from the TAIR database to identify the candidate reference genes of *P*. *praeruptorum* in different treatments. Then, a local BLAST program was conducted to obtain the sequences. The gene name, description, *Arabidopsis* homolog locus, amplification length combined with TBLASTN score and E-value of the twelve genes were listed in Table 1. The identity (ID), from 59% to 96%, indicated that they had a good homology to the selected protein sequences. To prove the specificity of the primers designed in this study, the PCR products were detected with agarose gel electrophoresis and melting curve analysis was also conducted. As shown in S1 and S2 Figs, a single band and peak were observed, indicating that they had a good specificity. To calculate the amplification efficiency, the standard curve of the candidate genes was conducted using a 10-fold serial dilution of plasmid DNA containing given genes. According to the slope of the standard curve (S3 Fig), the PCR efficiency (E) and the regression coefficient (R^2^) were calculated and listed in Table 1 and S3 Fig, respectively. Briefly, the R^2^ for all the primers >0.99 and the E ranged from 92.34% to 109.23%.

### Expression profile of the reference genes

**Fig 1:**
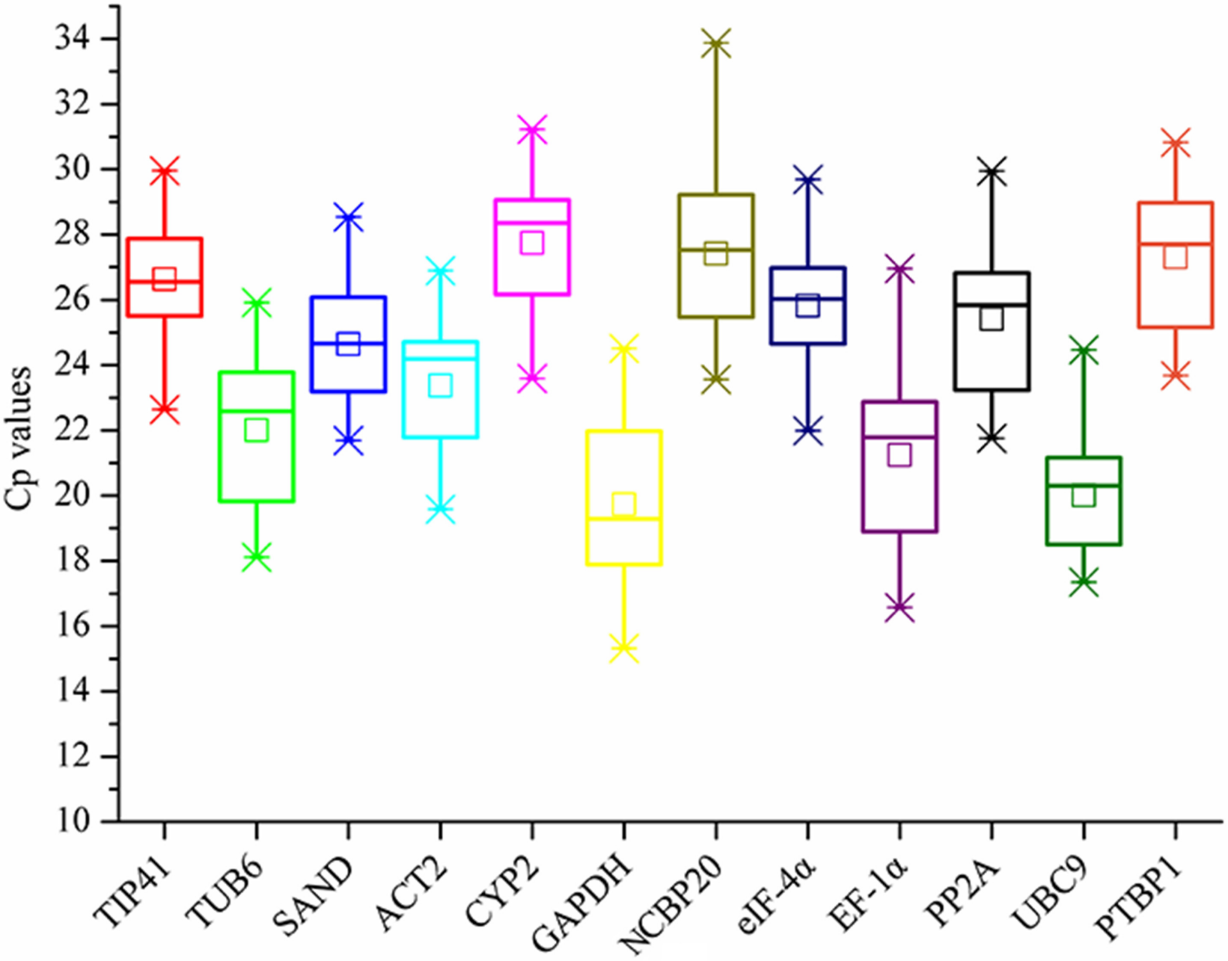
The comparison of transcript abundances of the twelve candidate reference genes. Boxes indicate the 25th/75th percentiles, the lines represent the median, squares represent the means and whiskers represent the maximum and minimum values.

The cycle threshold values (Cp) reflected the cycle number at which the fluorescence generated to the level that could be detected and low Cp values corresponded to high expression levels. Hence, expression profile of the reference genes could easily be seen in the form of Cp values. As shown in Fig 1, the mean Cp values for the twelve reference genes ranged from 19.73 to 27.74, indicating that they had an obvious difference in expression profile. In peculiar, majority of the values were distributed between 22 and 25. *GAPDH*, *EF-1α* and *UBC9* had low mean Cp values while *CYP2*, *BCBP20*, *PTBP1* tended to have higher Cp values. Among them, *GAPDH* had the lowest mean Cp value of 19.73 ± 2.269 while *CYP2* had the highest mean Cp with 27.74 ± 1.61. The figure also included gene expression variation in the selected genes and all treated samples with box-plot. Significantly, SAND showed low variability with a narrow distribution range of Cp values from 21.69 to 28.54, indicating that it might have a stable expression under different treatments and could be chosen as the best reference gene. While, *EF-1α*, with the Cp values differing from 16.57 to 26.96, might not be a good choice as a reference gene. In general, Cp values, combined with box-plot, could not only display the expression profile of the reference genes, but also give us a glimpse in gene stability. However, considering the complex surroundings of the plants, the stability of reference genes in different treatments needs to be investigated systematically.

### Expression stability of candidate reference genes

In order to further evaluate the stability of candidate reference genes under a given treatment, the selected twelve reference genes were supposed to different treatments (osmotic stress, salt stress, oxidative stress, heavy metal stress, hormone and temperature) and tissues, and then, 1188 Cp values (three biological and technical replicates) were collected for data analysis with three Excel-based programs (geNorm [24], NormFinder [23] and BestKeeper [35]).

### geNorm analysis

**Fig 2:**
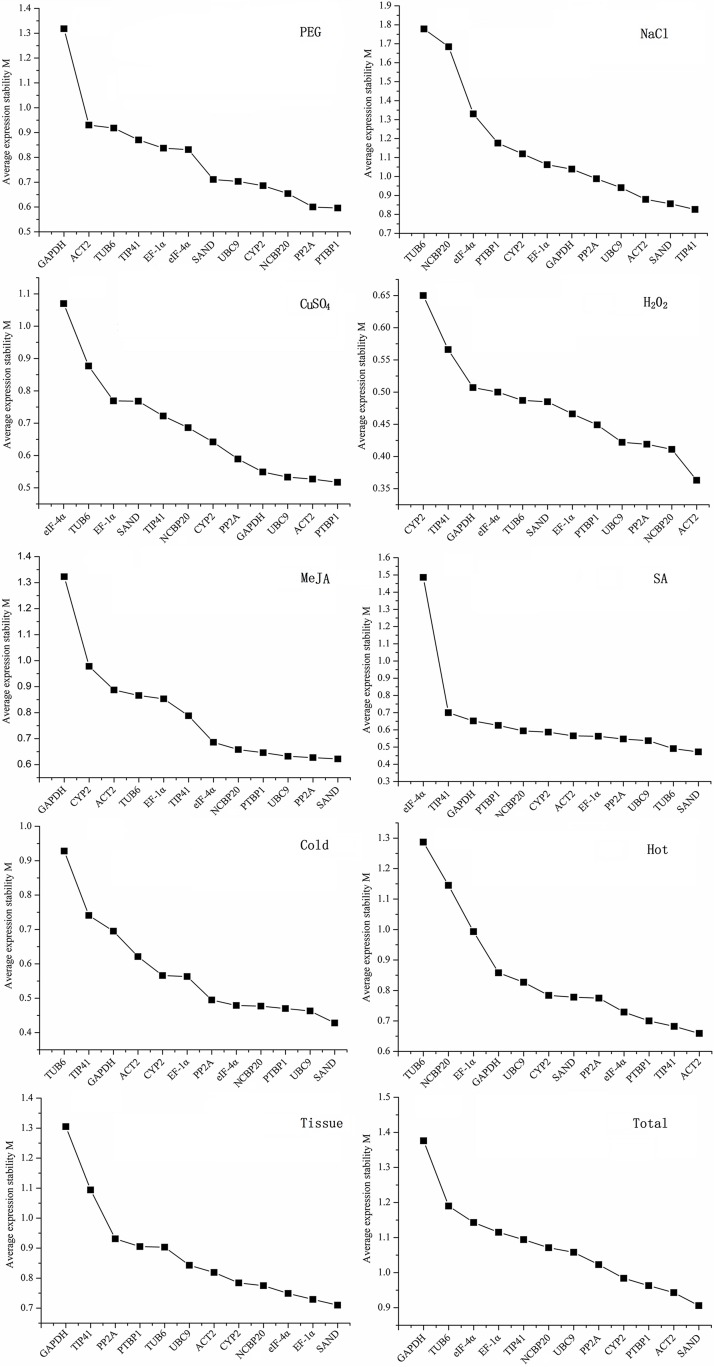
Expression stability of twelve candidate genes in *P*. *praeruptorum* as predicted by geNorm analysis. Average expression stability (M) in each treatment is calculated. The least stable gene which has a high M value is on the left, and the most stable gene on the right. The methods of treatment or group classification are listed in the picture correspondingly.

geNorm analysis uses the reference gene expression stability measurement (M) value which is calculated as the level of pairwise variation for each reference gene with all other control genes and as the standard deviation (SD) of the logarithmically transformed expression ratios to evaluate the gene expression stability and a high M value means a low stability [24]. To collect data for geNorm analysis, the plant was exposed to different treatments and the Cp values were processed via a linear scale using the ΔCp method [24]. As shown in Fig 2, different reference genes had different stability. For instance, PTBP1 was a most stable gene in drought treatment, while *GAPDH* had least stability in the same treatment. More interestingly, the same reference genes seemed to have different stability under different treatments. TIP41 ranked the most stable reference gene in NaCl induced salt stress, while, in the condition of H_2_O_2_ addition, a cold environment and different tissues, it tended to be one of the least stable reference gene. Another important information we could find was that, H_2_O_2_ had a least effect on the expression of reference genes with a low span of M value (0.35 to 0.65), while, when it came to NaCl or SA, the span was enlarged to 1. In another word, although *CYP2* was not a good reference gene in H_2_O_2_ induced oxidative stress, it might also be used as a reference gene for its low M value. To be the most important findings, *SAND* seemed to be a most stable reference gene under different stresses (5 times to be the most stable genes in 9 stresses or 10 groups) and could be selected as a good reference gene in different experimental designs.

### NormFinder analysis

NormFinder is an algorithm to identify the optimal normalization gene in a given experimental design. Similar to geNorm, the data from qRT-PCR run should not be used directly and needs to be transformed [23]. After data collection and analysis, the results of the gene stability ranking were shown in Table 2. As it was revealed, the rank of stability values gradually increased, which represented that the stability gradually decreased from top to bottom of the table. Hence, it could easily be seen that, *PTBP1*, *UBC9*, *ACT2* and *SAND* ranked the most stable reference genes in the treatment of PEG, CuSO_4_, H_2_O_2_ (hot) and NaCl (MeJA, SA, cold), respectively. Among the most stable reference genes, *SAND* had a lowest value which may be considered as the most important reference genes. More interestingly, *SAND* ranked nearly on the top of all the treatments (6 out of 10 groups) with which similar to the outcomes of geNorm analysis (Fig 2). However, there are also slightly differences between the results of geNorm and NormFinder analysis. For instance, *PTBP1* and *ACT2* were considered as the most stable reference genes by geNorm (Fig 2), while they ranked second and forth by NormFinder (Table 2), respectively. Hence, another analysis method needed to be involved.

**Table 2:** Expression stability values of twelve reference genes as calculated by the NormFinder in *P*. *praeruptorum*.

Rank	PEG	NaCl	CuSO_4_	H_2_O_2_	MeJA	SA	Cold	Hot	Tissue	Total
**1**	*PTBP1*	*SAND*	*UBC9*	*ACT2*	*SAND*	*SAND*	*SAND*	*ACT2*	*SAND*	*SAND*
	0.17	0.061	0.167	0.076	0.215	0.085	0.091	0.242098	0.288	0.463
**2**	*PP2A*	*TIP41*	*PTBP1*	*NCBP20*	*PP2A*	*TUB6*	*PTBP1*	*PTBP1*	*EF-1α*	*ACT2*
	0.189	0.15	0.193	0.215	0.253	0.18	0.197	0.263187	0.367	0.556
**3**	*NCBP20*	*ACT2*	*GAPDH*	*UBC9*	*UBC9*	*UBC9*	*UBC9*	*TIP41*	*NCBP20*	*PTBP1*
	0.349	0.245	0.221	0.238	0.308	0.243	0.22	0.340158	0.435	0.566
**4**	*CYP2*	*UBC9*	*ACT2*	*PP2A*	*PTBP1*	*PP2A*	*NCBP20*	*PP2A*	*eIF-4α*	*CYP2*
	0.357	0.539	0.234	0.25	0.315	0.275	0.231	0.456	0.44	0.622
**5**	*UBC9*	*PP2A*	*PP2A*	*PTBP1*	*NCBP20*	*EF-1α*	*eIF-4α*	*eIF-4α*	*CYP2*	*PP2A*
	0.372	0.61	0.329	0.308	0.35	0.331	0.237	0.472	0.494	0.677
**6**	*SAND*	*GAPDH*	*CYP2*	*EF-1α*	*eIF-4α*	*CYP2*	*PP2A*	*CYP2*	*ACT2*	*UBC9*
	0.467	0.681	0.439	0.328	0.363	0.362	0.26	0.479	0.568	0.746
**7**	*EF-1α*	*EF-1α*	*NCBP20*	*SAND*	*TIP41*	*ACT2*	*EF-1α*	*UBC9*	*UBC9*	*NCBP20*
	0.595	0.737	0.502	0.364	0.56	0.375	0.399	0.544	0.611	0.76
**8**	*TIP41*	*CYP2*	*TIP41*	*TUB6*	*EF-1α*	*NCBP20*	*CYP2*	*SAND*	*PTBP1*	*TIP41*
	0.643	0.758	0.528	0.368	0.659	0.4	0.409	0.55	0.643	0.785
**9**	*eIF-4α*	*PTBP1*	*SAND*	*eIF-4α*	*TUB6*	*PTBP1*	*ACT2*	*GAPDH*	*TUB6*	*EF-1α*
	0.649	0.837	0.616	0.37	0.7	0.428	0.49	0.668	0.655	0.832
**10**	*TUB6*	*eIF-4α*	*EF-1α*	*GAPDH*	*ACT2*	*GAPDH*	*GAPDH*	*EF-1α*	*PP2A*	*eIF-4α*
	0.725	1.09	0.627	0.383	0.727	0.486	0.595	0.862	0.692	0.873
**11**	*ACT2*	*NCBP20*	*TUB6*	*TIP41*	*CYP2*	*TIP41*	*TIP41*	*NCBP20*	*TIP41*	*TUB6*
	0.792	1.607	0.742	0.485	0.804	0.512	0.634	1.021	0.907	0.932
**12**	*GAPDH*	*TUB6*	*eIF-4α*	*CYP2*	*GAPDH*	*eIF-4α*	*TUB6*	*TUB6*	*GAPDH*	*GAPDH*
	1.234	1.686	0.981	0.578	1.253	1.444	0.857	1.183	1.179	1.18

### BestKeeper analysis

BestKeeper is an Excel-based tool using pairwise correlations to determine the stability of housekeeping genes, differentially regulated target genes and sample integrity [35]. The coefficient of variance (CV) and the standard deviation (SD) of the candidate reference genes were used to evaluate the stability of reference genes in each experimental design and the gene with the lowest CV and SD was considered to be the most stable reference gene [47]. It differs from the geNorm and NormFinder analysis and could use the raw data of Cp values to analyze. Similar to the results of NormFinder analysis, the CV ± SD rank of the candidate genes gradually increased from top to bottom of the table, which represented that the stability gradually decreased. For instance, *CYP2* had a CV ± SD value of 1.83 ± 0.53 and ranked as the most stable genes in PEG induced osmotic stress, while, *GAPDH* was listed as the least stable gene for it had a CV ± SD value of 7.40 ± 1.51 (Table 3). Owing to the fact that SD>1 was considered as inconsistent and such values should be excluded [41], none of the reference genes could be used in the group of ‘total’ for the least SD = 1.5. Fortunately, in another 9 groups or experimental designs, nearly all SD values were below 1.5 except the most unstable one. Some reference genes, namely *CYP2*, *ACT2* and *TIP41*, had a tendency to be the most stable genes for they were listed on top 3 of the ranks. On the contrary, *TUB6* and *EF-1α* seemed not to be good reference genes.

**Table 3:** Expression stability values of twelve reference genes as calculated by the BestKepper in *P*. *praeruptorum*.

Rank	PEG	NaCl	CuSO_4_	H_2_O_2_	MeJA	SA	Cold	Hot	Tissue	Total
**1**	*CYP2*	*TUB6*	*PTBP1*	*TUB6*	*TIP41*	*TIP41*	*PTBP1*	*PP2A*	*ACT2*	*eIF-4α*
	1.83±0.53	1.76±0.39	1.02±0.28	0.86±0.16	2.67±0.67	1.31±0.34	1.15±0.28	1.52±0.40	1.54±0.38	5.85±1.50
**2**	*ACT2*	*eIF-4α*	*ACT2*	*PTBP1*	*GAPDH*	*PTBP1*	*TIP41*	*PTBP1*	*PTBP1*	*TIP41*
	1.93±0.46	2.67±0.69	1.05±0.26	0.89±0.21	3.01±0.53	2.19±0.56	1.51±0.39	1.75±0.51	1.98±0.57	6.32±1.67
**3**	*PP2A*	*CYP2*	*PP2A*	*NCBP20*	*NCBP20*	*TUB6*	*PP2A*	*NCBP20*	*CYP2*	*PTBP1*
	2.10±0.54	3.23±0.92	1.18±0.32	1.39±0.34	3.43±0.89	2.20±0.44	1.60±0.37	1.78±0.52	2.07±0.60	6.42±1.75
**4**	*NCBP20*	*PTBP1*	*CYP2*	*SAND*	*eIF-4α*	*SAND*	*SAND*	*UBC9*	*eIF-4α*	*UBC9*
	2.10±0.58	3.38±0.95	1.50±0.44	1.51±0.34	3.78±0.94	2.29±0.52	1.72±0.40	1.87±0.40	2.53±0.68	6.73±1.35
**5**	*SAND*	*TIP41*	*NCBP20*	*ACT2*	*PTBP1*	*PP2A*	*eIF-4α*	*ACT2*	*NCBP20*	*CYP2*
	2.27±0.56	4.17±1.12	1.59±0.45	1.57±0.31	4.33±1.11	2.32±0.54	1.76±0.44	2.21±0.57	2.67±0.78	6.83±1.88
**6**	*eIF-4α*	*ACT2*	*UBC9*	*PP2A*	*SAND*	*CYP2*	*NCBP20*	*TIP41*	*SAND*	*SAND*
	2.29±0.60	4.51±1.09	1.67±0.35	1.69±0.38	4.56±1.07	2.59±0.65	1.81±0.46	2.53±0.71	2.71±0.70	6.86±1.68
**7**	*PTBP1*	*SAND*	*SAND*	*UBC9*	*PP2A*	*UBC9*	*UBC9*	*eIF-4α*	*EF-1α*	*NCBP20*
	2.40±0.67	4.7±1.18	2.14±0.54	1.94±0.35	5.02±1.20	3.05±0.57	2.20±0.40	3.16±0.85	2.93±0.66	7.52±2.05
**8**	*UBC9*	*PP2A*	*TIP41*	*TIP41*	*CYP2*	*NCBP20*	*CYP2*	*SAND*	*PP2A*	*PP2A*
	3.67±0.77	5.69±1.5	2.23±0.61	1.98±0.47	5.71±1.50	3.08±0.76	2.62±0.69	3.18±0.86	3.12±0.85	7.62±1.92
**9**	*TIP41*	*EF-1α*	*GAPDH*	*CYP2*	*UBC9*	*GAPDH*	*ACT2*	*TUB6*	*UBC9*	*ACT2*
	3.69±1.01	7.22±1.64	2.30±0.44	2.23±0.58	5.87±1.10	3.15±0.58	3.00±0.65	3.32±0.79	3.35±0.69	8.24±1.91
**10**	*EF-1α*	*GAPDH*	*EF-1α*	*eIF-4α*	*TUB6*	*ACT2*	*EF-1α*	*CYP2*	*TUB6*	*TUB6*
	3.72±0.84	7.46±1.46	2.66±0.59	2.43±0.56	7.54±1.55	3.34±0.71	3.19±0.62	3.45±1.01	3.76±0.88	9.52±2.08
**11**	*TUB6*	*UBC9*	*eIF-4α*	*EF-1α*	*ACT2*	*EF-1α*	*TUB6*	*GAPDH*	*TIP41*	*EF-1α*
	4.45±1.05	7.64±1.59	3.05±0.82	2.49±0.43	7.69±1.68	3.73±0.68	3.38±0.65	4.46±0.98	3.79±1.05	9.96±2.10
**12**	*GAPDH*	*NCBP20*	*TUB6*	*GAPDH*	*EF-1α*	*eIF-4α*	*GAPDH*	*EF-1α*	*GAPDH*	*GAPDH*
	7.40±1.51	8.18±2.32	3.14±0.74	2.96±0.49	7.77±1.53	4.41±1.08	4.29±0.76	5.04±1.21	4.68±1.03	10.34±2.0

### Optimal numbers of reference genes for accurate normalization

**Fig 3:**
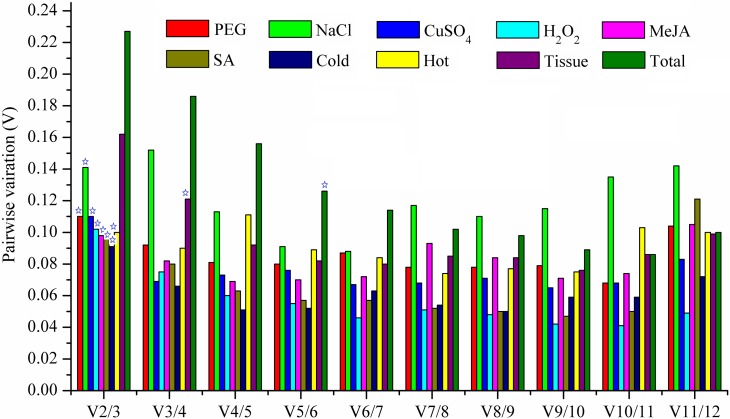
Determination of the optimal numbers of reference genes for normalization by pairwise variation using geNorm. Pairwise variation (Vn/n+1) analysis between the normalization factors (NFn and NFn+1) was performed in all treated samples. Different treatments are included and markerd as square frame with different colors. The total group refers to all samples. The V (variation) value with which below 0.15 was labeled with asterisk, representing that addition of a further reference gene does not result in any improvement of normalization.

Apart from using average pairwise expression ratios (M) to evaluate the gene expression stability, geNorm can also determine the optimal numbers of reference genes for normalization by calculating the pairwise variation (Vn/Vn+1) between the normalization factors (NF) in all samples of the different experimental sets using 0.15 as the proposed cut-off value [24]. According to this principle, the Vn/Vn+1 value was calculated and listed in Fig 3. As indicated, the two most stable reference genes were sufficient for reliable normalization under all treatments and there was no need to add a new reference gene in the treatment. However, when the experimental design related to different tissues, choosing three reference genes were necessary since V2/3>0.15. The same settlements were also suited for the total samples. Despite the fact that adding a reference gene might make it credible in qPCR analysis, the proposed 0.15 value must not be taken as a too strict cut off in most time, simply using the two best reference genes were reliable enough for normalization [24], and the results of this study also proved this idea.

### Reference gene validation

**Fig 4:**
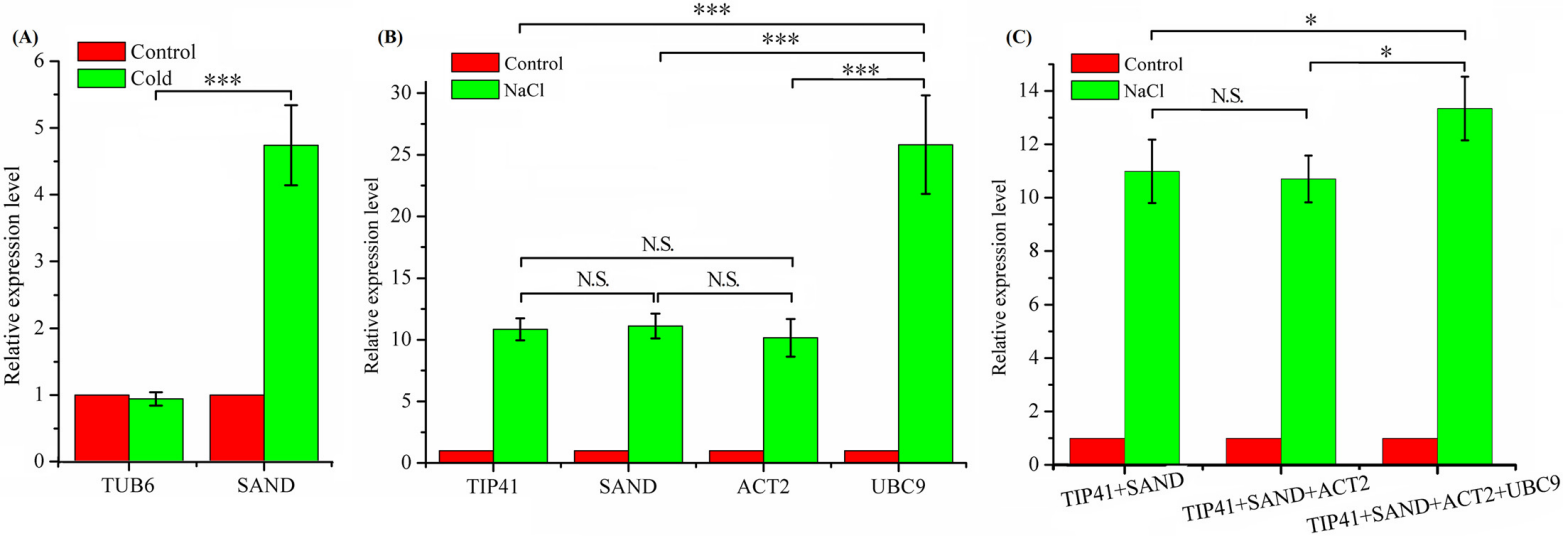
Validation of the reference genes. Relative expression level of *ACO* was normalized using candidate reference genes under different treatments. (A) Expression level was normalized using most and least stable reference genes under cold treatment. (B) Expression level was normalized using different reference genes under NaCl treatment. *TIP41*, *SAND* and *ACT2* represent the three most stable reference genes and *UBC9* is a least stable gene. (C) Expression level was normalized using different combination. Data are displayed as means ± SEM, and the statistical analyses were performed using the Student’s t-test to compare those between two reference genes or combination for normalization. *P< 0.05; ***P< 0.001; N.S.: No significant difference.

To evaluate the reliability of the selected reference genes, the relative expression level of *ACO* was calculated by the selected reference genes. As depicted in Fig 4A, an enhanced expression level of *ACO* was observed when normalized with the most stable reference gene, *SAND*. While, when it came to *TUB6*, one of the least reference genes, no obvious change was observed. To further evaluate the reliability of the selected reference genes, another stimulus was imposed and the three most stable reference genes were used to analyze the expression level of *ACO* at the same time. The results displayed that the expression level of *ACO* was enhanced at a same level (no significant difference) and all the three stable reference genes were reliable for normalization (Fig 4B). However, a significant difference (*P*<0.001) was observed when using *UBC9*, one of the most unstable reference genes. According to the results of geNorm, the optimal numbers of reference genes used for normalization was also investigated. The results showed that, though *UBC9* was not a good reference gene, the outcomes for normalization became advisable when it was combined with other stable genes (Fig 4C).

## Discussion

Considering the high sensitivity and specificity, qPCR is now commonly used in many laboratories for high-throughput analysis of gene transcript level. And, the utilization of suitable reference genes is necessary to ensure the reliability and accuracy of the data. With the awareness of the importance of reference genes in normalization and the deviation aroused by selection of the unstable reference genes, numerous studies have been conducted to investigate the stability of reference genes under different stresses or experimental designs [22,38,39]. Hence, in this study, the expression stability of twelve candidate reference genes in *P*. *praeruptorum* was systematically analyzed by geNorm, NormFinder, and BestKeeper under the treatment of osmotic stress (PEG), salt stress (NaCl), oxidative stress (H_2_O_2_), mental stress (CuSO_4_), hormones (MeJA and SA), cold (4°C) and hot (42°C) stress, and different tissue types. The results indicated that different reference genes needed to be selected under the different stresses since they shared different stabilities.

At the first step of the study, the twelve reference genes were cloned from the cDNA template and then inserted into T-Vector for sequencing. The correctness of the genes was confirmed by multiple sequence alignment with our transcriptome data (S3 Table). PCR was also conducted with the genomic DNA as a template and the products were inserted into T-Vector for sequencing, which could ensure the rationality of the primer design. As shown in S1 Fig, the signal band indicated the specificity of the primers while the difference of the band displayed the length of products with different templates. At the same time, the melting curve analysis was conducted to confirm the specificity of the primer pairs (S2 Fig). Moreover, the amplification efficiency of the selected candidate genes was calculated based on the slope of the standard curve. The R^2^ >0.99 and E-value ranged from 92.34% to 109.23% (Table 1 and S3 Fig), indicating that the curves showed a good linear relationship and the PCR conditions were acceptable.

As an important part of reference gene selection, the expression level of the selected genes was also investigated and the mean Cp values were listed in Fig 1. It can be easily seen that, the mean average expression level ranged from 19.73 to 27.74, which was consistent with the previous studies [18,41]. Based on the fact that the moderate expression level (a Cp value of 15 to 30, for instance) could give an accurate normalization [48], the genes selected in this study were sufficient for experimental needs. According to the fact that, low Cp values corresponded to high expression levels, some candidate genes selected in this study were abundantly distributed in *P*. *praeruptorum*. For instance, the *GAPDH* has a mean Cp value about 17 in *P*. *praeruptorum*, while the same value was up to 27 in carrot [40]. Considering that a narrow distribution range tends to represent the low variability, the variation of Cp values indicates that *SAND* is a best reference gene and *EF-1α* is the least one. The results were somehow consistent with the outcomes calculated by geNorm and NorFinder (Fig 2 and Table 2). Hence, considering the difference in the stability and the expression level of the candidate reference genes, the stability analysis and the expression analysis need to be combined.

In an ongoing effort to be more accurate in analyzing the stability of the candidate genes, three Excell-based programs were used according to the reports previously [23,24,35,49]. Owing to the fact that different software had its own method to rank the stability of the candidate genes and there might be some extent of disunity in the results, it was necessary to choose at least two methods to analyze the data. In addition, considering the reference genes had different stability under different treatments, osmotic stress, salt stress, oxidative stress, mental stress, hormones, temperature and different tissue types was imposed. The treatments conducted in the study nearly included all the treatments in similar studies and would present a systematically assess in gene stability [22,30,40,50].

With the help of the three analysis methods, the stability of the candidate genes was ranked in their own way of calculation. According to the results of geNorm analysis, *SAND* and *ACT2* were the two most stable reference genes in total samples. The outcomes were consistent with the results of NormFinder but against the outcomes of BestKeeper, in which *eIF-4α* and *TIP41* were the most stable reference genes with a lower CV. The same event also appeared in the treatment of PEG, CuSO_4_, H_2_O_2_, MeJA, SA and temperature stress or different tissue patterns. The results accounted for the fact that, geNorm and NormFinder analysis used the same way of calculation while BestKeeper using CV ± SD to rank the stability. This phenomenon was also reported by Zhuang and Tian in their studies [22,40]. However, it tended to be a good consistency when the five most stable reference genes were compared. For instance, under the treatment of PEG induced osmotic stress, the rank orders in geNorm, NormFinder and BestKeeper analysis were *PTBP1*>*PP2A*>*NCBP20*>*CYP2*>*UBC9*, *PTBP1*>*PP2A*>*NCBP20*>*CYP2*>*UBC9* and *CYP2*>*ACT2*>*PP2A*>*NCBP20*>*SAND*, respectively. In fact, there were no obvious differences in the order of the top five most stable reference genes. It also can be seen in Fig 4B, when normalized with *TIP41*, *ACT2*, and *SAND*, there were no significance differences in the expression level of *ACO*. Hence, it is sufficient to predict the stability of the reference genes by combination of the three kinds of software, which is a good strategy for the selection of reference genes for normalization [51,52]. For example, when merging the results with the three kinds of software, *SAND*, *ACT2*, *UBC9 PP2A* and *PTBP1* were the most stable reference genes identified under different treatments and they could be easily found on the top list of Fig 2, Tables 2 and 3. One of them could be chosen as the best reference gene in different stress experiments and the results were consistent with the previous studies [53–55]. However, the candidate genes with low stability could also be used for normalization. For instance, although *GAPDH* nearly ranked in the last of the candidate genes, it could also be used in MeJA induced stress experiment since it had a low CV value and the highest expression abundance. There were also numerous studies indicating that, *GAPDH* was among the most stable genes and usually used for measuring gene expression [34,56–59]. Hence, to be mentioned, stress and expression abundance shared equal importance in choosing a suitable reference gene.

The results above gave us enough information in reference gene selection under different treatments. A suitable reference gene could be chosen in qPCR analysis according to different experimental designs or treatments. However, the question that how many reference genes needed to be used in a specific set aroused. To resolve this problem, geNorm was employed according to the handbook which known as the ‘pairwise variation (V)’ [24]. They also recommended a V score of 0.15 as an ideal cut-off value, below which the inclusion of an additional reference gene was not required [24]. Based on the guidance, the optimal numbers of reference genes were calculated and listed in Fig 3. In our experiments, 8 out of 10 groups had a V score of below 0.15, indicating that there was no need to add an additional reference gene in those 8 groups. The results were also proved in Fig 4B where there were no visible differences between two or three reference genes for normalization. The same results were also proved by the study of Gimeno *et al*, in which different combination of reference genes was used for normalization of the target genes [39]. However, when the V score exceeded 0.15, it was desirable to add one more reference gene. As shown in Fig 4C, when *UBC9*, one of least stability reference genes, combined with other stable genes, the outcomes for normalization seemed to be credible. It also indicated that the proposed 0.15 value must not be taken as a too strict cut-off [24]. This was indeed true for several reports using a higher V values in some species according to the research purpose [48,60].

## Conclusions

qRT-PCR is the most commonly used method for gene expression analysis and a suitable reference gene is necessary for normalization. In the present study, the stability of twelve candidate reference genes in *P*. *praeruptorum* was investigated to select the most stable reference gene under different treatments. The stability analysis of gene expression by geNorm, NormFinder and BestKepper revealed that *SAND*, *ACT2*, *UBC9*, *PP2A* and *PTBP1* were the most stable reference genes which could be used for normalization. However, *GAPDH* and *TUB6* were the least stable genes. The optimal numbers of reference genes for normalization were also calculated by geNorm using the pairwise variation (Vn/Vn+1) and the results indicated that, in most of the treatments, two most stable reference genes were sufficient for normalization. In addition, the suitability of the most stable reference genes and their combination were confirmed by normalizing the expression of *ACO*. Apart from updating the first survey of the stability of reference genes in *P*. *praeruptorum* and providing the basis for further research in *P*. *praeruptorum*, the study also provided guidelines to obtain more accurate qRT-PCR results in other plant species.

## Supporting Information

S1 Fig Agarose gel (1%) electrophresis of the twelve candidate reference genes. 1–12 represent *TIP41*, *TUB6*, *SAND*, *ACT2*, *CYP2*, *GAPDH*, *NCBP20*, *eIF-4α*, *EF-1α*, *PP2A*, *UBC9*, *PTBP1*, respectively. The left part is the PCR products with cDNA as template and the right part is the PCR products with gDNA as template.
(TIF)

S2 Fig Melt curves of the twelve candidate reference genes. (TIF)

S3 Fig Standard curves of twelve candidate reference genes. (TIF)

S1 Table Raw Cp values in *P*. *praeruptorum*. Plants were subjected to the following stress treatments: PEG, NaCl, CuSO_4_, H_2_O_2_, MeJA, cold, hot, SA and different tissues.
(DOCX)

S2 Table Nucleotide acid sequences of twelve candidate reference genes from *P*. *praeruptorum*. The PCR was conducted with the cDNA and genomic DNA as templates and then the products were inserted into T-Vector for sequencing, respectively. 1–12 represent the twelve candidate reference genes and the sequences with the italics represents the intron of each gene.
(DOCX)

S3 Table The transcriptome data of twelve candidate reference genes. (XLSX)

## References

[1] Kumar A, Maurya R, Sharma S, Ahmad P, Singh A, Bhatia G, et al Pyranocoumarins: a new class of anti-hyperglycemic and anti-dyslipidemic agents. Bioorg Med Chem Lett. 2009; 19: 6447–6451. 10.1016/j.bmcl.2009.09.031 19811915

[2] Wu J, Fong W, Zhang J, Leung C, Kwong H, Yang M, et al Reversal of multidrug resistance in cancer cells by pyranocoumarins isolated from Radix Peucedani. Eur J Pharmacol. 2003; 473: 9–17. 12877932 10.1016/s0014-2999(03)01946-0

[3] Yu P, Jin H, Zhang J, Wang G, Li J, Zhu Z, et al Pyranocoumarins isolated from Peucedanum praeruptorum Dunn suppress lipopolysaccharide-induced inflammatory response in murine macrophages through inhibition of NF-κB and STAT3 activation. Inflammation. 2012; 35: 967–977. 10.1007/s10753-011-9400-y 22083490

[4] Hou Z, Luo J, Wang J, Kong L. Separation of minor coumarins from Peucedanum praeruptorum using HSCCC and preparative HPLC guided by HPLC/MS. Sep Purif Technol. 2010; 75: 132–137.

[5] Ling-Yi K, Yi L, Zhi-Da M, Xian L, Ting-Ru Z. Coumarins from Peucedanum praeruptorum. Phytochemistry. 1996; 41: 1423–1426.

[6] Lin Y, Sun X, Yuan Q, Yan Y. Combinatorial biosynthesis of plant-specific coumarins in bacteria. Metab Eng. 2013; 18: 69–77. 10.1016/j.ymben.2013.04.004 23644174

[7] Paddon CJ, Westfall P, Pitera D, Benjamin K, Fisher K, Mcphee D, et al High-level semi-synthetic production of the potent antimalarial artemisinin. Nature. 2013; 496: 528–532. 10.1038/nature12051 23575629

[8] Qu Y, Easson ML, Froese J, Simionescu R, Hudlicky T, Luca VD. Completion of the seven-step pathway from tabersonine to the anticancer drug precursor vindoline and its assembly in yeast. P Natl Acad Sci USA. 2015; 112: 6224–6229. 10.1073/pnas.1501821112 PMC4434687 25918424

[9] Bourgaud F, Hehn A, Larbat R, Doerper S, Gontier E, Kellner S, et al Biosynthesis of coumarins in plants: a major pathway still to be unravelled for cytochrome P450 enzymes. Phytochem Rev. 2006; 5: 293–308.

[10] Kawai Y, Ono E, Mizutani M. Evolution and diversity of the 2-oxoglutarate-dependent dioxygenase superfamily in plants. Plant J. 2014; 78: 328–343. 10.1111/tpj.12479 24547750

[11] Larbat R, Hehn A, Hans J, Schneider S, Jugde H, Schneider B, et al Isolation and functional characterization of CYP71AJ4 encoding for the first P450 monooxygenase of angular furanocoumarin biosynthesis. J Biol Chem. 2009; 284: 4776–4785. 10.1074/jbc.M807351200 19098286

[12] Karamat F, Olry A, Munakata R, Koeduka T, Sugiyama A, Paris C, et al A coumarin-specific prenyltransferase catalyzes the crucial biosynthetic reaction for furanocoumarin formation in parsley. Plant J. 2014; 77: 627–638. 10.1111/tpj.12409 24354545

[13] Ishikawa A, Kuma T, Sasaki H, Sasaki N, Ozeki Y, Kobayashi N, et al Constitutive expression of bergaptol O-methyltransferase in Glehnia littoralis cell cultures. Plant Cell Rep. 2009; 28: 257–265. 10.1007/s00299-008-0631-9 18974989

[14] Zhao Y, Liu T, Luo J, Zhang Q, Xu S, Han C, et al Integration of a decrescent transcriptome and metabolomics dataset of Peucedanum praeruptorum to investigate the CYP450 and MDR genes involved in coumarins biosynthesis and transport. Front Plant Sci. 2015; 6: 996 10.3389/fpls.2015.00996 26697023 PMC4674560

[15] Wan XL, Zhou Q, Wang YY, Wang WE, Bao MZ, Zhang JW. Identification of heat-responsive genes in carnation (Dianthus caryophyllus L.) by RNA-seq. Front Plant Sci. 2015; 6: 519 10.3389/fpls.2015.00519 26236320 PMC4500917

[16] Li C, Wang Y, Huang X, Li J, Wang H, Li J. An improved fruit transcriptome and the identification of the candidate genes involved in fruit abscission induced by carbohydrate stress in litchi. Front Plant Sci. 2015; 6: 439 10.3389/fpls.2015.00439 26124768 PMC4466451

[17] Sun Y, Luo H, Li Y, Sun C, Song J, Niu Y, et al Pyrosequencing of the Camptotheca acuminata transcriptome reveals putative genes involved in camptothecin biosynthesis and transport. BMC Genomics. 2011; 12: 533 10.1186/1471-2164-12-533 22035094 PMC3229617

[18] Li X, Zhang D, Li H, Gao B, Yang H, Zhang Y, et al Characterization of reference genes for RT-qPCR in the desert moss Syntrichia caninervis in response to abiotic stress and desiccation/rehydration. Front Plant Sci. 2015; 6: 38 10.3389/fpls.2015.00038 25699066 PMC4318276

[19] Li D, Ono N, Sato T, Sugiura T, Altaf-Ul-Amin M, Ohta D, et al Targeted Integration of RNA-Seq and Metabolite Data to Elucidate Curcuminoid Biosynthesis in Four Curcuma Species. Plant Cell Physiol. 2015; 56(5):843–851. 10.1093/pcp/pcv008 25637373

[20] Caldana C, Scheible WR, Mueller-Roeber B, Ruzicic S. A quantitative RT-PCR platform for high-throughput expression profiling of 2500 rice transcription factors. Plant Methods. 2007; 3: 7 17559651 10.1186/1746-4811-3-7 PMC1914063

[21] Bustin SA, Benes V, Garson JA, Hellemans J, Huggett J, Kubista M, et al The MIQE guidelines: minimum information for publication of quantitative real-time PCR experiments. Clin Chem. 2009; 55: 611–622. 10.1373/clinchem.2008.112797 19246619

[22] Zhuang H, Fu Y, He W, Wang L, Wei Y. Selection of appropriate reference genes for quantitative real-time PCR in Oxytropis ochrocephala Bunge using transcriptome datasets under abiotic stress treatments. Front Plant Sci. 2015; 6: 475 10.3389/fpls.2015.00475 26175743 PMC4484982

[23] Andersen CL, Jensen JL, Ørntoft TF. Normalization of real-time quantitative reverse transcription-PCR data: a model-based variance estimation approach to identify genes suited for normalization, applied to bladder and colon cancer data sets. Cancer Res. 2004; 64: 5245–5250. 15289330 10.1158/0008-5472.CAN-04-0496

[24] Vandesompele J, De Preter K, Pattyn F, Poppe B, Van Roy N, Paepe A, et al Accurate normalization of real-time quantitative RT-PCR data by geometric averaging of multiple internal control genes. Genome Biol. 2002; 3: 7. 10.1186/gb-2002-3-7-research0034 PMC126239 12184808

[25] Schmittgen TD, Zakrajsek BA. Effect of experimental treatment on housekeeping gene expression: validation by real-time, quantitative RT-PCR. J Biochem Bioph Meth. 2000; 46: 69–81. 10.1016/s0165-022x(00)00129-9 11086195

[26] Ruan W, Lai M. Actin, a reliable marker of internal control? Clin Chim Acta. 2007; 385: 1–5. 17698053 10.1016/j.cca.2007.07.003

[27] Czechowski T, Stitt M, Altmann T, Udvardi MK, Scheible W-R. Genome-wide identification and testing of superior reference genes for transcript normalization in Arabidopsis. Plant Physiol. 2005; 139: 5–17. 16166256 10.1104/pp.105.063743 PMC1203353

[28] Wang H, Yang B, Geng T, Li B, Dai P, Chen C, et al Tissue-specific selection of optimal reference genes for expression analysis of anti-cancer drug-related genes in tumor samples using quantitative real-time RT-PCR. Exp Mol Pathol. 2015; 98: 375–381. 10.1016/j.yexmp.2014.10.014 25445497

[29] Leal MF, Astur DC, Debieux P, Arliani GG, Franciozi CES, Loyola LC, et al Identification of Suitable Reference Genes for Investigating Gene Expression in Anterior Cruciate Ligament Injury by Using Reverse Transcription-Quantitative PCR. PLoS One. 2015; 10: e0133323 10.1371/journal.pone.0133323 26192306 PMC4507999

[30] Yuan M, Lu Y, Zhu X, Wan H, Shakeel M, Zhan S, et al Selection and evaluation of potential reference genes for gene expression analysis in the brown planthopper, Nilaparvata lugens (Hemiptera: Delphacidae) using reverse-transcription quantitative PCR. PLoS One. 2014; 9: e86503 10.1371/journal.pone.0086503 24466124 PMC3900570

[31] Cusick KD, Fitzgerald LA, Cockrell AL, Biffinger JC. Selection and Evaluation of Reference Genes for Reverse Transcription-Quantitative PCR Expression Studies in a Thermophilic Bacterium Grown under Different Culture Conditions. PLoS One. 2015; 10: e0131015 10.1371/journal.pone.0131015 26115538 PMC4482720

[32] Verbeke J, Van Poucke M, Peelman L, De Vliegher S. Differential expression of CXCR1 and commonly used reference genes in bovine milk somatic cells following experimental intramammary challenge. BMC Genet. 2015; 16: 40 10.1186/s12863-015-0197-9 25895496 PMC4421990

[33] Pollier J, Bossche RV, Rischer H, Goossens A. Selection and validation of reference genes for transcript normalization in gene expression studies in Catharanthus roseus. Plant Physiol Bioch. 2014; 83: 20–25. 10.1016/j.plaphy.2014.07.004 25058454

[34] Jain M, Nijhawan A, Tyagi AK, Khurana JP. Validation of housekeeping genes as internal control for studying gene expression in rice by quantitative real-time PCR. Biochem Bioph Res Co. 2006; 345: 646–651. 10.1016/j.bbrc.2006.04.140 16690022

[35] Pfaffl MW, Tichopad A, Prgomet C, Neuvians TP. Determination of stable housekeeping genes, differentially regulated target genes and sample integrity: BestKeeper-Excel-based tool using pair-wise correlations. Biotechnol Lett. 2004; 26: 509–515. 15127793 10.1023/b:bile.0000019559.84305.47

[36] Xie F, Xiao P, Chen D, Xu L, Zhang B. miRDeepFinder: a miRNA analysis tool for deep sequencing of plant small RNAs. Plant Mol Biol. 2012; 80: 75–84. 10.1007/s11103-012-9885-2 22290409

[37] Silver N, Best S, Jiang J, Thein SL. Selection of housekeeping genes for gene expression studies in human reticulocytes using real-time PCR. BMC Mol Biol. 2006; 7: 33 17026756 10.1186/1471-2199-7-33 PMC1609175

[38] Jiang Q, Wang F, Li M-Y, Ma J, Tan G-F, Xiong A-S, et al Selection of suitable reference genes for qPCR normalization under abiotic stresses in Oenanthe javanica (BI.) DC. PLoS One. 2014; 9: e92262 10.1371/journal.pone.0092262 24651080 PMC3961309

[39] Gimeno J, Eattock N, Van Deynze A, Blumwald E. Selection and validation of reference genes for gene expression analysis in switchgrass (Panicum virgatum) using quantitative real-time RT-PCR. PLoS One. 2014; 9: e91474 10.1371/journal.pone.0091474 24621568 PMC3951385

[40] Tian C, Jiang Q, Wang F, Wang G-L, Xu Z-S, Xiong A-S, et al Selection of suitable reference genes for qPCR normalization under abiotic stresses and hormone stimuli in carrot leaves. PLoS One. 2015; 10(2): e0117569 10.1371/journal.pone.0117569 25658122 PMC4319972

[41] Lin Y, Zhang C, Lan H, Gao S, Liu H, Liu J, et al Validation of potential reference genes for qPCR in maize across abiotic stresses, hormone treatments, and tissue types. PLoS One. 2014; 9(5): e95445 10.1371/journal.pone.0095445 24810581 PMC4014480

[42] Nakashima A, Tanimura-Ito K, Oshiro N, Eguchi S, Miyamoto T, Momonami A, et al A positive role of mammalian Tip41-like protein, TIPRL, in the amino-acid dependent mTORC1-signaling pathway through interaction with PP2A. FEBS Lett. 2013; 587: 2924–2929. 10.1016/j.febslet.2013.07.027 23892082

[43] Ling H, Wu Q, Guo J, Xu L, Que Y. Comprehensive selection of reference genes for gene expression normalization in sugarcane by real time quantitative RT-PCR. PLoS One. 2014; 9(5): e97469 10.1371/journal.pone.0097469 24823940 PMC4019594

[44] Borowski JM, Galli V, da Silva Messias R, Perin EC, Buss JH, Silva SDA, et al Selection of candidate reference genes for real-time PCR studies in lettuce under abiotic stresses. Planta. 2014; 239: 1187–1200. 10.1007/s00425-014-2041-2 24573225

[45] Nicot N, Hausman J-F, Hoffmann L, Evers D. Housekeeping gene selection for real-time RT-PCR normalization in potato during biotic and abiotic stress. J Exp Bot. 2005; 56: 2907–2914. 16188960 10.1093/jxb/eri285

[46] Pfaffl MW. A new mathematical model for relative quantification in real-time RT-PCR. Nucleic Acids Res. 2001; 29: e45–e45. 11328886 10.1093/nar/29.9.e45 PMC55695

[47] Xiao X, Ma J, Wang J, Wu X, Li P, Yao Y. Validation of suitable reference genes for gene expression analysis in the halophyte Salicornia europaea by real-time quantitative PCR. Front Plant Sci. 2014; 5:788 10.3389/fpls.2014.00788 25653658 PMC4300904

[48] Wan H, Zhao Z, Qian C, Sui Y, Malik AA, Chen J, et al Selection of appropriate reference genes for gene expression studies by quantitative real-time polymerase chain reaction in cucumber. Anal Biochem. 2010; 399: 257–261. 10.1016/j.ab.2009.12.008 20005862

[49] Huang L, Yan H, Jiang X, Yin G, Zhang X, Qi X, et al Identification of candidate reference genes in perennial ryegrass for quantitative RT-PCR under various abiotic stress conditions. PloS One. 2014; 9(4): e93724 10.1371/journal.pone.0093724 24699822 PMC3974806

[50] Lee JM, Roche JR, Donaghy DJ, Thrush A, Sathish P. Validation of reference genes for quantitative RT-PCR studies of gene expression in perennial ryegrass (Lolium perenne L.). BMC Mol Biol. 2010; 11: 8 10.1186/1471-2199-11-8 20089196 PMC2827471

[51] Paolacci AR, Tanzarella OA, Porceddu E, Ciaffi M. Identification and validation of reference genes for quantitative RT-PCR normalization in wheat. BMC Mol Biol.2009; 10: 11 10.1186/1471-2199-10-11 19232096 PMC2667184

[52] Reid KE, Olsson N, Schlosser J, Peng F, Lund ST. An optimized grapevine RNA isolation procedure and statistical determination of reference genes for real-time RT-PCR during berry development. BMC Plant Biol. 2006; 6: 27 17105665 10.1186/1471-2229-6-27 PMC1654153

[53] Migocka M, Papierniak A. Identification of suitable reference genes for studying gene expression in cucumber plants subjected to abiotic stress and growth regulators. Mol Breeding. 2011; 28: 343–357.

[54] Tong Z, Gao Z, Wang F, Zhou J, Zhang Z. Selection of reliable reference genes for gene expression studies in peach using real-time PCR. BMC Mol Biol. 2009; 10: 71 10.1186/1471-2199-10-71 19619301 PMC3224724

[55] Zhong H-Y, Chen J-W, Li C-Q, Chen L, Wu J-Y, Chen J-Y, et al Selection of reliable reference genes for expression studies by reverse transcription quantitative real-time PCR in litchi under different experimental conditions. Plant Cell Rep. 2011; 30: 641–653. 10.1007/s00299-010-0992-8 21301853

[56] Iskandar HM, Simpson RS, Casu RE, Bonnett GD, Maclean DJ, Manners JM. Comparison of reference genes for quantitative real-time polymerase chain reaction analysis of gene expression in sugarcane. Plant Mol Biol Rep.2004; 22: 325–337.

[57] Barsalobres-Cavallari CF, Severino FE, Maluf MP, Maia IG. Identification of suitable internal control genes for expression studies in Coffea arabica under different experimental conditions. BMC Mol Biol. 2009; 10: 1 10.1186/1471-2199-10-1 19126214 PMC2629470

[58] Li Q-F, Sun SS, Yuan D-Y, Yu H-X, Gu M-H, Gu M-H, et al Validation of candidate reference genes for the accurate normalization of real-time quantitative RT-PCR data in rice during seed development. Plant Mol Biol Rep. 2010; 28: 49–57.

[59] Podevin N, Krauss A, Henry I, Swennen R, Remy S. Selection and validation of reference genes for quantitative RT-PCR expression studies of the non-model crop Musa. Mol Breeding. 2012; 30: 1237–1252. 10.1007/s11032-012-9711-1 PMC3460175 23024595

[60] De Ketelaere A, Goossens K, Peelman L, Burvenich C. Technical note: validation of internal control genes for gene expression analysis in bovine polymorphonuclear leukocytes. J Dairy Sci. 2006; 89: 4066–4069. 16960083 10.3168/jds.S0022-0302(06)72450-X

