# Supplementary figures and images for: Selection of Reference Genes for Gene Expression Normalization in Peucedanum praeruptorum Dunn under Abiotic Stresses, Hormone Treatments and Different Tissues
Source: PLoS One. 2016 Mar 29;11(3):e0152356. doi: 10.1371/journal.pone.0152356 (PMC4811526; doi:10.1371/journal.pone.0152356)

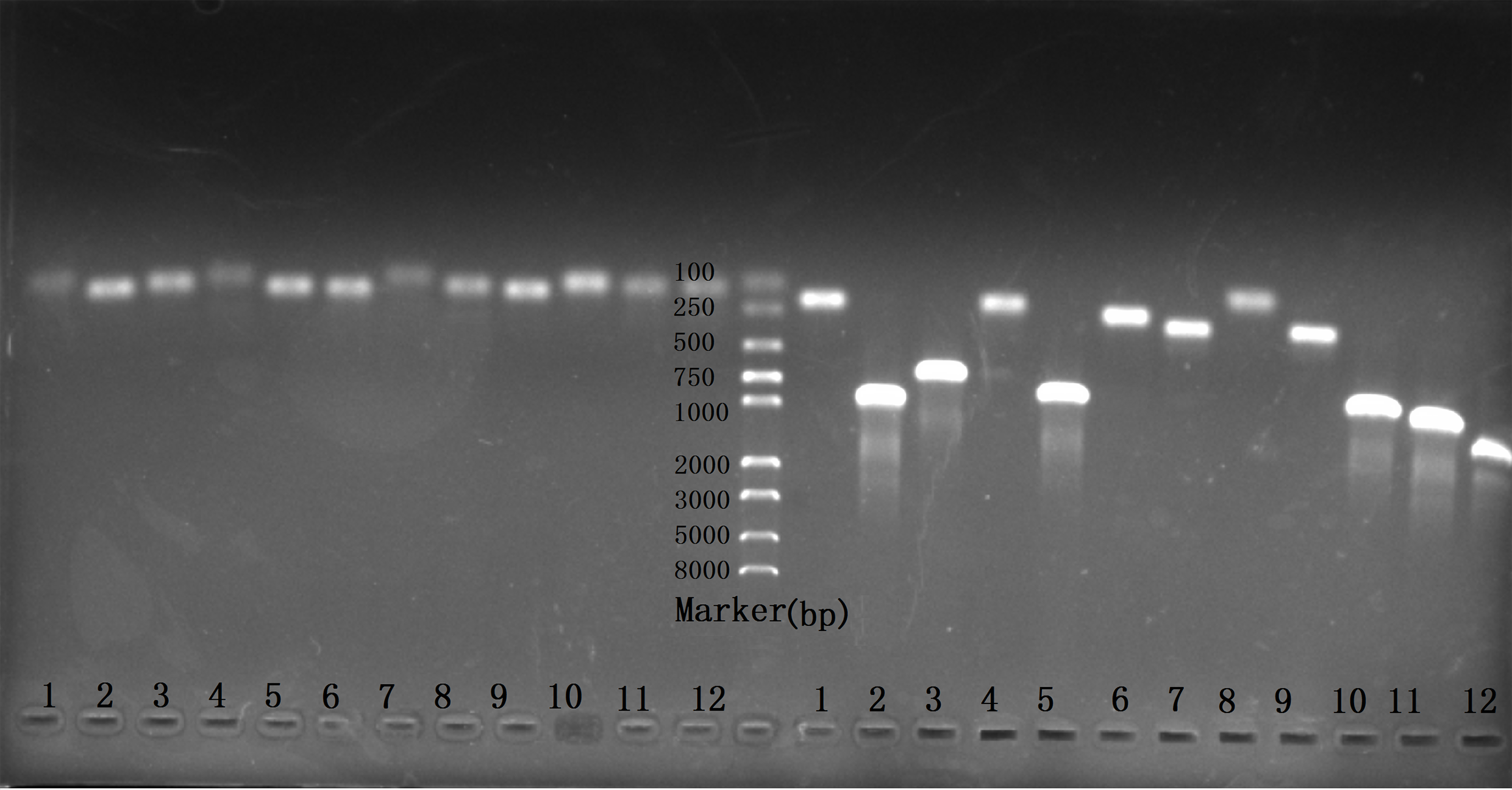

Supplement: S1 Fig — 1–12 represent TIP41, TUB6, SAND, ACT2, CYP2, GAPDH, NCBP20, eIF-4α, EF-1α, PP2A, UBC9, PTBP1, respectively. The left part is the PCR products with cDNA as template and the right part is the PCR products with gDNA as template. (TIF) [file pone.0152356.s001.tif]

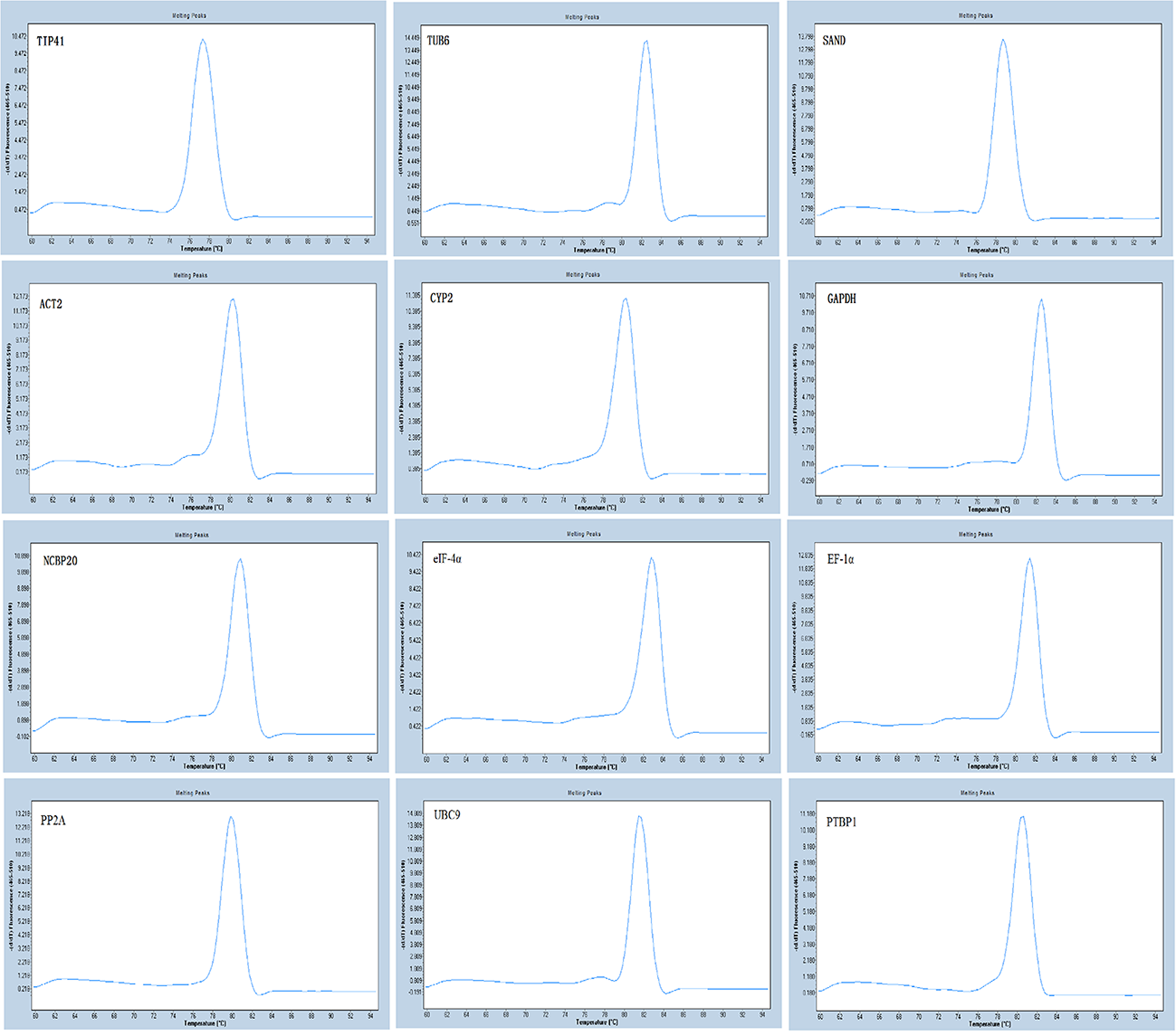

Supplement: S2 Fig — (TIF) [file pone.0152356.s002.tif]
