# Supplementary material for: Selection of Reference Genes for Gene Expression Normalization in Peucedanum praeruptorum Dunn under Abiotic Stresses, Hormone Treatments and Different Tissues
Source: PLoS One. 2016 Mar 29;11(3):e0152356. doi: 10.1371/journal.pone.0152356 (PMC4811526; doi:10.1371/journal.pone.0152356)
Supplement: S1 Table — Plants were subjected to the following stress treatments: PEG, NaCl, CuSO4, H2O2, MeJA, cold, hot, SA and different tissues. (DOCX) [file pone.0152356.s004.docx]

**Supplementary Information**

**S1 Table. Raw Cp values in *P. praeruptorum*.**

Plants were subjected to the following stress treatments: PEG, NaCl, CuSO_4_, H_2_O_2_, MeJA, cold, hot, SA and different tissues.

|  | ***TIP41*** | ***TUB6*** | ***SAND*** | ***ACT2*** | ***CYP2*** | ***GAPDH*** | ***NCBP20*** | ***eIF-4α*** | ***EF-1α*** | ***PP2A*** | ***UBC9*** | ***PTBP1*** |
| --- | --- | --- | --- | --- | --- | --- | --- | --- | --- | --- | --- | --- |
| **PEG** | 29.96 | 25.92 | 25.87 | 24.12 | 30.38 | 23.29 | 28.31 | 27.13 | 23.96 | 26.78 | 22.49 | 29.49 |
|  | 27.54 | 24.26 | 24.43 | 23.79 | 29.19 | 21.97 | 27.21 | 25.82 | 23.69 | 25.52 | 21.44 | 28.38 |
|  | 27.58 | 22.88 | 25.58 | 24.21 | 28.9 | 22.25 | 28.2 | 26.02 | 22.94 | 25.85 | 21.77 | 28.57 |
|  | 28.95 | 24.82 | 25.56 | 24.67 | 28.78 | 20.76 | 28.28 | 27.46 | 23 | 26.28 | 21.88 | 28.63 |
|  | 27.24 | 23.84 | 24.48 | 22.94 | 28.69 | 20 | 27.59 | 26.49 | 22.75 | 25.65 | 21.24 | 27.68 |
|  | 26.81 | 22.22 | 24.56 | 24.19 | 28.16 | 19.76 | 26.99 | 25.35 | 21.65 | 24.75 | 21.37 | 27.76 |
|  | 27.16 | 24.07 | 25 | 24.44 | 28.96 | 19.25 | 27.12 | 26.41 | 21.47 | 25.5 | 20.45 | 27.9 |
|  | 25.63 | 22.8 | 23.95 | 23.5 | 27.85 | 17.89 | 26.79 | 26.44 | 22.36 | 24.93 | 19.93 | 26.97 |
|  | 25.48 | 21.9 | 24.38 | 23.33 | 28.01 | 18.17 | 26.53 | 25.09 | 20.8 | 24.58 | 19.49 | 26.71 |
| **NaCl** | 25.68 | 22.41 | 24.13 | 22.87 | 27.4 | 18.27 | 26.31 | 25.38 | 20.84 | 24.9 | 19.32 | 28.89 |
|  | 29.77 | 22.85 | 27.63 | 26.14 | 29.4 | 22.58 | 33.87 | 27.57 | 26.96 | 29.94 | 24.47 | 30.43 |
|  | 29.21 | 21.97 | 27.69 | 26.87 | 31.22 | 22.93 | 33.4 | 26.44 | 25.84 | 29.45 | 24.26 | 29.63 |
|  | 26.91 | 23.28 | 25.25 | 23.55 | 29.06 | 18.59 | 27.44 | 26.53 | 22.29 | 24.88 | 20.3 | 28.04 |
|  | 25.92 | 22.4 | 23.82 | 22.89 | 27.74 | 17.45 | 26.67 | 25.66 | 21.68 | 24.93 | 19.21 | 27.07 |
|  | 25.85 | 21.85 | 24.3 | 22.79 | 27.6 | 17.68 | 26.39 | 25.38 | 21.15 | 25.01 | 19.53 | 27.52 |
|  | 26.22 | 22.58 | 23.92 | 23.64 | 27.95 | 19.32 | 26.91 | 25.49 | 21.48 | 25.79 | 19.84 | 26.81 |
|  | 26.55 | 22.88 | 24.5 | 23.92 | 28.33 | 19.38 | 27.29 | 24.29 | 22.42 | 25.9 | 20.18 | 27.5 |
|  | 26.52 | 21.86 | 24.55 | 23.88 | 27.9 | 19.67 | 27.4 | 25.58 | 21.73 | 26.11 | 20.02 | 28.09 |
| **CuSO_4_** | 28.2 | 23.76 | 25.28 | 24.34 | 28.2 | 19.15 | 27.52 | 26.38 | 21.7 | 26.42 | 21.32 | 27.49 |
|  | 26.8 | 22.62 | 25.36 | 24.54 | 28.83 | 18.68 | 28.06 | 26.2 | 22.87 | 25.93 | 21.29 | 27.68 |
|  | 26.72 | 21.81 | 25.85 | 24.8 | 28.91 | 18.76 | 27.56 | 26.54 | 21.62 | 26.41 | 20.99 | 27.89 |
|  | 27.54 | 24.85 | 25.49 | 25.33 | 30.07 | 19.73 | 29.35 | 27.22 | 23.13 | 27.47 | 21.62 | 28.39 |
|  | 27.11 | 23.77 | 24.24 | 24.61 | 29.64 | 18.38 | 28.34 | 26.31 | 23.22 | 26.72 | 20.98 | 27.68 |
|  | 26.61 | 22.69 | 24.79 | 24.73 | 28.59 | 18.65 | 28.48 | 26.19 | 21.98 | 26.92 | 20.83 | 27.8 |
|  | 26.39 | 23.87 | 24.48 | 24.35 | 28.92 | 18.29 | 28.82 | 25.99 | 21.86 | 26.44 | 20.67 | 27.39 |
|  | 27.87 | 23.7 | 26.34 | 24.71 | 28.95 | 19.45 | 28.33 | 29.69 | 21.86 | 26.76 | 21.82 | 27.94 |
|  | 28.21 | 24.24 | 25.92 | 25.26 | 29.44 | 19.41 | 28.84 | 27.43 | 22.8 | 26.82 | 21.88 | 28.52 |
| **H_2_O_2_** | 24.33 | 18.86 | 22.74 | 20.73 | 25.19 | 17.43 | 25.26 | 23.97 | 18.41 | 22.91 | 18.14 | 24.4 |
|  | 24.39 | 18.74 | 22.16 | 20.38 | 24.7 | 16.72 | 24.91 | 23.52 | 17.82 | 22.79 | 18.32 | 23.73 |
|  | 23.56 | 18.45 | 21.98 | 19.85 | 23.91 | 16.31 | 24.31 | 22.71 | 17.32 | 22.33 | 17.38 | 23.68 |
|  | 23.08 | 18.41 | 21.69 | 19.89 | 25.22 | 16.79 | 24.33 | 22.51 | 17.64 | 21.82 | 17.51 | 23.71 |
|  | 23.15 | 18.81 | 22.65 | 19.86 | 24.92 | 16.38 | 24.44 | 22.74 | 16.95 | 21.91 | 17.87 | 24 |
|  | 23.48 | 18.54 | 21.7 | 19.69 | 23.87 | 15.44 | 24.05 | 21.99 | 16.78 | 21.75 | 17.39 | 23.71 |
|  | 23.7 | 18.46 | 21.87 | 20.24 | 24.22 | 16.5 | 23.89 | 22.9 | 17.21 | 22.54 | 17.61 | 23.69 |
|  | 22.64 | 18.56 | 22.32 | 19.58 | 24.11 | 15.32 | 23.78 | 22.26 | 16.57 | 21.75 | 17.55 | 23.98 |
|  | 22.93 | 18.24 | 22.53 | 20.29 | 25.94 | 16.79 | 24.28 | 23.82 | 17.11 | 22.27 | 18.42 | 24.15 |
| **MeJA** | 24.75 | 19.32 | 22.62 | 20.82 | 26.59 | 19.28 | 25.62 | 25.2 | 19.36 | 23.56 | 18.25 | 25.38 |
|  | 24.93 | 19.34 | 23.24 | 21.41 | 26.17 | 18.88 | 25.47 | 24.55 | 18.89 | 22.84 | 17.71 | 25.13 |
|  | 24.26 | 18.92 | 22.29 | 20.3 | 24.58 | 17.67 | 24.39 | 23.54 | 18.54 | 22.86 | 17.35 | 24.1 |
|  | 24.55 | 19.95 | 22.81 | 20.42 | 23.75 | 17.54 | 25.74 | 24.66 | 17.99 | 23.43 | 18.38 | 25.28 |
|  | 24.34 | 19.63 | 22.77 | 20.34 | 25.64 | 17.36 | 25.49 | 24.15 | 18.56 | 23.1 | 18.36 | 25.16 |
|  | 23.63 | 19.27 | 22.33 | 19.98 | 24.3 | 16.67 | 24.91 | 23.37 | 17.73 | 22.33 | 17.64 | 24.39 |
|  | 25.81 | 23.41 | 25.18 | 24.35 | 28.52 | 18.9 | 27.35 | 26.3 | 22.71 | 25.84 | 20.49 | 27.26 |
|  | 26.4 | 23.38 | 25.32 | 24.7 | 28.88 | 18.77 | 27.75 | 26.79 | 21.64 | 25.91 | 20.71 | 27.44 |
|  | 25.48 | 21.9 | 24.76 | 23.9 | 27.7 | 17.37 | 26.72 | 25.54 | 21.49 | 25.42 | 20.09 | 27.54 |
| **Cold** | 25.99 | 18.97 | 23.52 | 22.11 | 27.51 | 18.91 | 25.54 | 25.67 | 20.55 | 23.43 | 18.97 | 25.32 |
|  | 26.57 | 19.3 | 23.43 | 21.9 | 27.33 | 19.13 | 25.93 | 25.51 | 20.54 | 23.18 | 18.49 | 25.08 |
|  | 26.47 | 18.96 | 23.27 | 21.65 | 26.8 | 18.39 | 25.17 | 24.83 | 20.22 | 22.95 | 18.33 | 24.94 |
|  | 25.58 | 19.93 | 23.19 | 21.81 | 26.85 | 17.61 | 25.25 | 25.44 | 19.52 | 23.75 | 18.8 | 24.75 |
|  | 25.51 | 20.42 | 23.37 | 22.82 | 27.39 | 17.34 | 25.9 | 24.97 | 19.4 | 23.17 | 18.66 | 24.88 |
|  | 24.83 | 19.79 | 23.28 | 22.2 | 26.53 | 17.12 | 25.37 | 24.62 | 19.47 | 23 | 18.56 | 24.56 |
|  | 25.67 | 18.11 | 22.49 | 20.81 | 25.83 | 16.76 | 24.53 | 24.65 | 18.73 | 22.4 | 17.7 | 24.35 |
|  | 25.57 | 18.42 | 22.61 | 20.73 | 25.65 | 17 | 24.78 | 24.29 | 18.54 | 22.29 | 18 | 24.3 |
|  | 25.86 | 20.16 | 22.25 | 20.33 | 25.07 | 16.8 | 24.22 | 24.17 | 18.59 | 22.6 | 17.49 | 24.46 |
| **Hot** | 29.44 | 23.78 | 28.54 | 26.61 | 30.73 | 23.41 | 29.49 | 28.74 | 25.52 | 26.7 | 21.79 | 30.08 |
|  | 29.4 | 23.54 | 27.99 | 26.89 | 30.56 | 22.7 | 28.45 | 28.22 | 25.98 | 26.45 | 21.69 | 29.61 |
|  | 28.77 | 22.58 | 27.94 | 26.78 | 29.73 | 23.5 | 28.65 | 27.89 | 25.34 | 27.24 | 20.9 | 29.2 |
|  | 28.18 | 25.58 | 26.79 | 25.89 | 30.2 | 21.8 | 30.97 | 26.97 | 23.86 | 26.5 | 21.78 | 29.9 |
|  | 27.79 | 24.85 | 26.33 | 25.81 | 29.8 | 22.25 | 29.4 | 26.72 | 24.8 | 26.58 | 21.72 | 29.28 |
|  | 27.78 | 22.75 | 26.21 | 25.75 | 28.72 | 21.65 | 29.71 | 26.54 | 23.55 | 26.59 | 21.54 | 29.43 |
|  | 27.47 | 24.35 | 25.99 | 25.33 | 28.62 | 21.15 | 29.11 | 26.62 | 22.87 | 25.9 | 21.49 | 28.49 |
|  | 27.55 | 23.73 | 25.96 | 25.14 | 27.71 | 19.95 | 29.22 | 25.71 | 22.7 | 25.55 | 20.52 | 28.56 |
|  | 26.94 | 22.61 | 26.11 | 24.92 | 27.59 | 20.39 | 28.75 | 25.61 | 21.8 | 25.87 | 20.89 | 28.26 |
| **SA** | 25.78 | 19.27 | 21.81 | 20.28 | 24.43 | 17.31 | 23.68 | 23.9 | 17.59 | 22.72 | 17.66 | 24.85 |
|  | 25.21 | 19.25 | 21.89 | 20.24 | 24.5 | 17.59 | 23.65 | 23.68 | 17 | 23.23 | 17.58 | 24.8 |
|  | 26.13 | 19.99 | 22.64 | 21.75 | 25.11 | 18.35 | 24.9 | 24.8 | 18.7 | 23.82 | 18.99 | 25.93 |
|  | 25.32 | 19.66 | 22.68 | 21.37 | 25.78 | 19.2 | 24.92 | 24.24 | 18.72 | 23.66 | 18.5 | 25.78 |
|  | 25.49 | 19.82 | 22.65 | 21.81 | 25.24 | 18.92 | 25.29 | 23.64 | 18.13 | 23.17 | 18.79 | 24.93 |
|  | 25.86 | 20.95 | 23.95 | 22.67 | 26.27 | 19.19 | 25.95 | 24.8 | 19.27 | 24.65 | 19.78 | 26.48 |
|  | 26.3 | 20.43 | 23.45 | 21.99 | 25.77 | 18.63 | 25.72 | 28.98 | 18.81 | 24.25 | 19.65 | 25.91 |
|  | 25.11 | 19.13 | 21.83 | 20.35 | 23.59 | 17.97 | 23.56 | 23.48 | 16.89 | 22.95 | 18.38 | 25.27 |
|  | 25.55 | 19.87 | 22.58 | 21.78 | 24.76 | 17.88 | 25.3 | 23.58 | 18.37 | 22.89 | 18.63 | 24.64 |
| **Tissue** | 28.33 | 24.34 | 25.88 | 24.42 | 28.42 | 24.51 | 29.97 | 27.2 | 23.15 | 27.92 | 20.43 | 29.34 |
|  | 28 | 24.51 | 26.33 | 24.5 | 28.94 | 22.12 | 29.51 | 27.71 | 22.25 | 27.21 | 20.89 | 29.34 |
|  | 28.53 | 24.32 | 26.26 | 24.52 | 28.81 | 23.91 | 29.98 | 27.49 | 22.13 | 27.22 | 20.78 | 29.33 |
|  | 28.02 | 24.58 | 26.34 | 24.54 | 28.97 | 22.85 | 29.74 | 27.23 | 22.43 | 27.05 | 20.9 | 29.55 |
|  | 28.27 | 24.62 | 26.6 | 24.55 | 29.13 | 22.44 | 29.72 | 26.9 | 22.33 | 27.1 | 21.01 | 29.49 |
|  | 29.12 | 23.73 | 26.77 | 24.95 | 29.68 | 22.64 | 29.65 | 27.83 | 23.5 | 28.43 | 21.16 | 29.87 |
|  | 29.74 | 23.87 | 26.69 | 24.94 | 29.21 | 23.72 | 30.07 | 27.63 | 23.21 | 28 | 21.05 | 29.78 |
|  | 29.44 | 23.81 | 26.92 | 24.87 | 29.82 | 22.12 | 29.65 | 27.23 | 23.64 | 27.87 | 21.02 | 29.8 |
|  | 29.03 | 23.71 | 26.68 | 25.11 | 29.9 | 22.79 | 29.73 | 27.6 | 23.42 | 27.85 | 20.6 | 29.52 |
|  | 27.47 | 23.76 | 26.15 | 24.53 | 29.43 | 21.88 | 29.31 | 27.15 | 22.63 | 27.55 | 20.31 | 29.2 |
|  | 27.45 | 23.77 | 25.72 | 24.22 | 28.68 | 21.75 | 28.65 | 25.79 | 22.45 | 27.57 | 20.3 | 28.71 |
|  | 27.61 | 24.26 | 26.21 | 24.75 | 29.29 | 21.88 | 29.74 | 26.94 | 22.29 | 26.63 | 20.14 | 30.82 |
|  | 28.75 | 24.63 | 27.5 | 26.71 | 30.54 | 22.3 | 30.76 | 28.96 | 23.94 | 28.17 | 22.77 | 29.78 |
|  | 29.86 | 24.92 | 26.28 | 24.46 | 30.98 | 23.32 | 30.91 | 28 | 23.08 | 28.86 | 21.88 | 29.37 |
|  | 29.62 | 23.84 | 26.41 | 24.31 | 28.53 | 22.75 | 30.67 | 27.5 | 23.85 | 29.14 | 21.71 | 29.51 |
|  | 25.42 | 22.93 | 26.44 | 24.81 | 29.65 | 22.45 | 29.69 | 27.52 | 22.89 | 27.97 | 21.05 | 28.84 |
|  | 26.42 | 21.77 | 24.89 | 24.35 | 27.74 | 20.42 | 27.88 | 26.39 | 21.41 | 25.95 | 19.95 | 27.7 |
|  | 25.94 | 22.27 | 25.82 | 24.48 | 28.92 | 22.18 | 29.3 | 26.77 | 22.48 | 27.76 | 20.67 | 28.98 |
|  | 25.67 | 22.48 | 24.58 | 24.33 | 28.53 | 19.66 | 27.74 | 26.45 | 21.78 | 25.42 | 20.33 | 28.09 |
|  | 26.4 | 22.64 | 24.76 | 24.49 | 28.35 | 19.7 | 27.97 | 26.51 | 21.77 | 25.84 | 20.25 | 27.62 |
|  | 25.43 | 21.62 | 23.81 | 22.6 | 26.7 | 22.79 | 26.94 | 24.13 | 19.76 | 24.53 | 17.58 | 28.8 |
|  | 27.54 | 24.2 | 24.98 | 24.94 | 29.13 | 19.26 | 29.19 | 27.19 | 21.86 | 26.67 | 21.45 | 28.01 |
|  | 27.32 | 23.84 | 24.66 | 24.66 | 28.96 | 19.88 | 28.95 | 26.8 | 22.53 | 26.72 | 20.9 | 27.69 |
|  | 27.43 | 21.46 | 24.61 | 23.48 | 27.96 | 21.28 | 28.29 | 25.36 | 21.21 | 26.45 | 18.64 | 28.95 |
|  | 28.7 | 22.31 | 25.88 | 24.63 | 28.75 | 21.76 | 27.45 | 26.3 | 22.33 | 29.17 | 20.89 | 28.97 |
|  | 27.88 | 22.3 | 25.48 | 23.93 | 28.68 | 20 | 28.87 | 26.03 | 21.81 | 27.43 | 19.2 | 28.92 |
|  | 26.95 | 22.9 | 26.08 | 24.82 | 29.4 | 21.98 | 29.4 | 26.57 | 22.77 | 28.22 | 21.46 | 28.85 |
