# Supplementary material for: Selection of Reference Genes for Gene Expression Normalization in Peucedanum praeruptorum Dunn under Abiotic Stresses, Hormone Treatments and Different Tissues
Source: PLoS One. 2016 Mar 29;11(3):e0152356. doi: 10.1371/journal.pone.0152356 (PMC4811526; doi:10.1371/journal.pone.0152356)
Supplement: S2 Table — The PCR was conducted with the cDNA and genomic DNA as templates and then the products were inserted into T-Vector for sequencing, respectively. 1–12 represent the twelve candidate reference genes and the sequences with the italics represents the intron of each gene. (DOCX) [file pone.0152356.s005.docx]

**Supplementary Information**

**S2 Table. Nucleotide acid sequences of twelve candidate reference genes from *P. praeruptorum*.**

The PCR was conducted with the cDNA and genomic DNA as the template and then the products were inserted into T-Vector for sequencing, respectively. 1-12 represent the twelve candidate reference genes and the sequence with the italics represents the intron of each gene.

1 *TIP41:*

cDNA as template:

ttgactgcacttgcatcaaaagagcctattctcttctatgatgaggtaatcttctatgaagatgaattggctgatagtggagtgtcg

gDNA as template:

ttgactgcacttgcatcaaaagagcctattctcttctatgatgaggt*ttgtatttctaagaacatataatacatgggaacagatggaaaacaaaagttattacctctagtaggattgcttagtatatctttctttgtggtgacctaagctgttgtacttcaataggt*aatcttctatgaagacgaattggctgatagtggagtgtcg

2 *TUB6*:

cDNA as template:

ggtgctggtaataattgggccaaaggtcactatactgaaggtgctgagttgattgactcggtgcttgatgttgtcaggaaggaagctgagaattgtgactgtcttcaagggtttcaggtgtgtcattcacttggtggtggaactggatctggaatggg

gDNA as template:

ggtgctggtaataattgggcccaaaggtcactattactgaaggtgctgagttgattgactcggtgcttgatgttgtcaggaaggaagctgagaattgtgactgtcttcaagg*tctcctctccctccctccctctctctctctctctctctctctctctctctctctctctccctctctctctctctctctccctccctcactccccccctccctcactccctcactccctcactcactccctccctccctctctccctccccctctctctcttttatttgtagatctatgagtgttggtaatgtaaatgaacttgttgaaatgtgtctatattttgtttacatattctgatttttgtgtggtattttgttttattattgttgaagatgagatgactacagatctacgagtattggttatttatatgaagttgttgaaatgtttgtgtgtgtgtgtgcatagatctgcgggtattgacgtgtaaatgaacttgttgagatgtgtgtatgagataaggaagagtagtgtgtttacaaattgttttttgattgttattcacattacgtagtgctcgatctgtatccctttatgcctaggtttgtttattcagtatacaagtgtattatgtttgtgtctgatctgatttttatctacgtgatttgtttgttttattcagtctctaggtcactaactgtatgacagtaatgtatatacagatctgtatggatttaaacaagcatgtgattctccaatgttctgtatcggtgtcaattaactgagcacattattgtttgttatatgcaggtgtttgtctcatgtgtttcttcatgatttgtttttaaagatttatatgatcttgtgaggtcttgattatttttaatttgagaaattaatgctagacttgatatgacatgtgcgtatgtataagatttagatattgtgctgattattagtgatggttgtgcagggtttcagg*tgtgtcattcacttggtggtggaactggatctggaatggg

3 *SAND*:

cDNA as template:

acagaagagcctcatgaatccctcagtgaacaactggaacttctttatggccagatgctttgagaagaatccgaaatttgatatgacgcctttgcttg

gDNA as template:

acagaagagcctcatgaatccctcagtgaacaactggaacttctttatggccag*gtactccctccttcccacccaattatttacatttttctatttaggttatccctttggattctttacatttctaaaaaggagtaaagttttaatatcttaaagtttttcaacccactactttccttcagcccttttaaatttttgtactttattaaatattaattggaccactactttactcctttcttattctccagactcaacctatatgcagagtaatgggtggaggagtacaattcatctccatggctctatataagcttgagctattttagtaaaaatattttgtaccggaataagaaaacatagcatgttacattatttgttgagccaggagctcaagttatgattgttaaaggttatctgagctccttttaatcgagacagctttaagttagttgaattcaattgtaaccttcgagtgtagtcctggatattttatgacatcgctctctctctctctctctctctctctctctctctctaaaaacttatgtttttagttacacttgtgtgaaggggatagatggtagtttcttactgtttcaattcttataaatgcagatgatacttattctgacaaagtctataaatag*atgctttgagaagaatccgaaatttgatatgacgcctttgcttg

4 *ACT2*:

cDNA as template:

tttcactatatgccagtggtcgtacaactggtattgtgttggattctggtgatggtgttagtcacactgtcccgatctacgaag

gDNA as template:

tttcactatatgccagtggtcgtacaactggt*tcgtatctttctctcaaacatctgcaatttcgatttctatattgcttacggtccgtgtttgattattatatgttttaaaaatatgttacattttggtggttatttgcttcatttttttcccaggt*attgtgttggattctggtgatggtgttagtcacactgtcccgatctacgaag

5 *CYP2*:

cDNA as template:

cgttcagctctgtctcgaaggttattatgatgacacaatttttcatcgtataattaagtcatttatggtccaaggtggtgatcctactggcactggcaaaggtggtgaaagtatatatggaggtacattttctgatgagttccattcccgcctta

gDNA as template:

cgttcagctctgtctcgaaggttattatgatgacacaatttttcatcgtataattaagtcatttatggtccaaggtggtgatcctactggcactggcaaa*ggtaattttgtatgtcttttatgtgaattgaagttttgttgtgattgtttcgtgctggaattatcgaatatggtttaatttgagttagggtttgtgttttcgatacacgtttatctccgttgctctctagtttaatttggcatgtacggaattgagggaagaatgaggattacgaatatctagccattatagtacatatttcattcgaattaaaaaaaatttaaagtaattcattccttctaatcgtacggggcgttagggattagcgaaagctgggatatgtttaagtaggggatgccttatgatgcaaggggtaaaacttcgaggggtgtagattaaaatgatctagggaggacatgctggctggcaatgattttatgagtggttagactcctcaagttctataattctttgttggtcaatgtaccttatatctttgtaggaaactgccaattatgttagtcgagtacagattttaattattagcttctacactattaaacctgatggtagatctcttgtcccctatgcctgttatacaaacatctctatataaatacaattttgttttgcaatgtttgctagcttttgtgtgtattaattaatttgttttcactttgatgacacagaatattatgtagtagaatagttactatttcaaacaaatgcttgtcccctcatgatgtcattgatttgatgttcaca*ggtggtgaaagtatatatggaggtacatttttctgatgagttccattcccgcctta

6 *GAPDH*:

cDNA as template:

ggtcatgggagatgacatggtcaaggtcgtagcctggtacgataacgaatggggatacagccaaagagttgtagatttggcagatttggtagcaagcaaatggccaggtgcagctgtaactggaagtggtgattcattagaagatttttgcgagacaaaccctg

gDNA as template:

ggtcatgggagatgacatggtcaaggtcgtagcctggtacgataacgaatggggatacag*gtatgtgaagataatttaaaaaatggaagatgcattttcatgttctaatttgttgaggtgatcgtagtatctcattggagtgatattgtggttttgcag*ccaaagagttgtagatttggcagatttggtagcaagcaaatggccaggtgcagctgtaactggaagtggtgattcattagaagatttttgcgagacaaaccctg

7 *NCBP20:*

cDNA as template:

GGCCAGGTACGCGATGAATATCGCACCGACTATGATCCTGGCATGGGTGGTTATGGAAAATTAGTTCAGAAGGAGTTGGAAGCACAAAGGG

gDNA as template:

ggccaggtacgcgatgaatatcgcaccgactatgatcctg*atatccttttaagcacattacatgtttttcaattcctatttattctacttcctaaattattcgatcagtggcctaattttgatatatagattttagtacaactgatcagtagttcattaccaattaagattcaccgattatgatacttccagttattaatacccgtgaattctttcattaccaattaagttagttgaatctttagttttcctcaactagctgtac*gcatgggtggttatggaaaattagttcagaaggagttggaagcacaaaggg

8 *eIF-4α*:

cDNA as template:

gcgcagtcgtgaccacacagtttctgctactcatggagatatggatcagaacactagagacattatcatgagggaattcagatctggttcatctcgtgtgctcatcaccactgatctcctggcccgtggtattgatgtgcagcaa

gDNA as template:

gcgcagtcgtgaccacacagtttctgctactcatggagatatggatcagaacactagagacattatcatgagggaattcagatctggttcatctcgtgtgctcatcaccactgatctcctggcccgtggtattgatgtgcagcaa

9 *EF-1α*:

cDNA as template:

caagcagatgatctgttgctgcaacaagatggatgctacaacccccaagtactcgaagtctagatttgaagaaattgtgaaggaggtgtcttcttatttgaagaaggttgggtacaaccccgacaaaattgcactcattcccatctctggattcgagggtgacaacatga

gDNA as template:

caagcagatgatctgttgctgcaacaag*gtatagctttattacaagtttgttacaagttatatggtatctgttgtttactatggttttgtatctgtttatgtttaactatcctctgccaatgtatacaaagatctctttgatactgccttaaacttgttaataagtaataaattccacaaaaacattttttcaaggcgtttataacattttatattttgtattgtttaccttgtatag*atggatgctacaacccccaagtactcgaagtctagatttgaagaaattgtgaaggaggtgtcttcttatttgaagaaggttgggtacaaccccgacaaaattgcattcattcccatctctggattcgagggtgacaacatga

10 *PP2A*:

cDNA as template:

catggagggctttcaccatctctggatactttagataatatcagatccttggatcgtatccaagaggttccacatgaaggaccaatgtgtgatcttctatggtctgatccagacgaccg

gDNA as template:

catggagggctttcaccatctctggatactttagataatatcagatccttggatcgtatccaagaggt*acactaagtgaatagcatttggcatgtttgggattcgtagagcttgtggtgccatgtgtagtgtggttttatccaatcacaatactttatcatgcctattgttttcgtatgacaactttttcttacccttgcataaatttttgtatattcagtgcccccaacattgtgttcccgagtattttactcagaaaagaaaaaaaaattgaaaatacaaaggaaaaagaagaatttgtatacactttcacatgttcctgagtttttgttggaaacaaaggaaagcaagaatatgatgggataaagataaacgattacttttatatgtgtaagttttttttgtccaaatctttccacttttgggtgaaaattttcggaaagaaattgactctgcatttctcctattttcttttccttttgttctcttaaaaaaacttaggaatcaaaaaaaatattacatatccttctcttttctttgctctctaaaataaatcttggaaacacatcgtcaagatttgtacatatagttccctcactttaggaaaagtgggcatagatgagatctttgcatagctgcttcttcctcttcctgttttaattagacgtcaatagcgctcgaatatacctagggcaatgtatgtatggcatacgcttcttgtcaaccgtttatactcagtaatctcactttaagacctggttgggtacgcacatgtttcttacttttctgtgactgtctacatatgaatgctaggt*tccacatgaaggaccaatgtgtgatcttctatggtctgatccagacgaccg

11 *UBC9*：

cDNA as template:

ctcgaagcggatcttgaaggagctcaaggatctgcagaaagatcctcccacttcttgcagcgctggcccagttgccgaagacatgtttcattggcaagcaactatcatgggaccccctgacagtccatatgcgggtg

gDNA as template:

ctcgaagcggatcttgaaggagctcaaggatctgcagaaagatcctcccacttcttgcagcgctgg*tatttatattatcctactttgctatgtgtttttatatgctgtctctgtcataaagtttcgatttttattcttgttttcaggcttttaggttctttgttggtttaatattttatttaggttgttaatatgtttgaatttgattaattgtgagtgtagagttaggggattttgtgtgaaaccaaagtaatactagatgggttgctgttgaaacttgagattatgttgaacttaggctagtctttcggggcttggtagtcggttgtatttcgttgagattgtgagtatatatatgatttttgtatgtgggtcaccgtgcttactgaaaaaatgggcaatttggtttttttttatatgaagttgttcgatttgaagaaaggattataattgaaatgggctgtttgagctctcttttggttttattggataagctacttcagaaattttatctaattttgaggggtgggagagagactttattgcccgcacatgagtatcatgaacacggataagaatgaattagtcccaaaagagtattgggttttgctttactattgcacgtgcacatctatctctctcatttttcagggaagcacactcacacttgctcgagctcgtacacatataagcctaattcatgataatgtgtatgtgagatgaaattcatggttaacttggtgtcacaaacttgttcagttaccatttccctacatataggtagattataccattgggcataggctcacaagtgagtggtttatattagataccgagagccagctgtgggttggcatatgactgcattggcactattataacttgctgatttggtgctgcattttattcctgctatttatctccgttctcatatttttgcttttcggg*cccagttgccgaaggacatgtttcattggcaagcaactatcatggggaccccatgacagtccatatgcgggtg

12 *PTBP1*:

cDNA as template:

ccagaacatgttggttcttgcaatctgcgcatctcatattcagctcacacagatctaaacatcaagttccaatcacaccgtagccgggactatacaaatccatatcttcctgttaatgcaactgcaattgaggga

gDNA as template:

ccagaacatgttggttcttgcaatctgcgcatctcatattcagctcacacagatctaaacatcaagttccaatcacaccgtagccg*gtaatactgaatttacttggctactatatttcgtgtctccattgtgtatttagtcaggtcttatgcttgcctattatagtattatgccagtgcgttctcatttttgcagttgaggtcttgctaggggttagggaagctcggcagctaaaacagatttgtttttcatgtccacttagtaattttcttgatcttattgcgtgattttacctgcaccctagttgataccctagttgatggtttaagtgtgtatcagctgttggatgagctgtcctcaccatcctaattagtaaaacgagatgatagttgatttggtcctagtatttaccttgtgaaagggacatttactttctgcactgcatgcttcttgaacgagtttcttgaacactaagcttgactcgacctttacttaatttccatttctagatgcactaattacaagtcatgtgataattaaataatattatcagatattataaacttttttttcctctatttgtacaattaccttatagatatttgtttcaatgtgtacagttacattgcaaatataacatctttgtttgtgtgccaataaattgtaacacccatcacattagaatcactaatagcttattaactgattaaatgatgcattatatcatttggttatgtatatatgataaatgtcatgatctggtgtttattaaaaagttaaaggacttctttccttgatcttggagattgttatatatatgataaatgtcatgatctgttgtttattaaaaaataaatgacttctttcttaaatcttggagattgttactacaaattgctttctttcttgactgattactgtactccgtgaataggtgaatgtgcctaaagtttgtagcgtatgtaattcttcttcgttcatgtctcgttttacatcactgattaattttgtcaccacaaacttttttaaactcttaaagaaatagattgaggggcctggggggaagcacttgcttaattatatacgctcttcctttttttgagtcgctagttggctagtttttctatttttaatatcagttatctgtcttgcgcaattataattcattttctcgtcttacag*ggactatacaaatccatatctccctgttaatgcaactgcaattgaggga
